# Identification of Casz1 as a Regulatory Protein Controlling T Helper Cell Differentiation, Inflammation, and Immunity

**DOI:** 10.3389/fimmu.2018.00184

**Published:** 2018-02-07

**Authors:** Natarajan Bhaskaran, Zhihui Liu, Senthil S. Saravanamuthu, Chunhua Yan, Ying Hu, Lijin Dong, Peggy Zelenka, Lixin Zheng, Vassili Bletsos, Rachel Harris, Brenna Harrington, Aaron Weinberg, Carol J. Thiele, Fengchun Ye, Pushpa Pandiyan

**Affiliations:** ^1^Department of Biological Sciences, School of Dental Medicine, Case Western Reserve University, Cleveland, OH, United States; ^2^Cell and Molecular Biology Section, Pediatric Oncology Branch, National Cancer Institute, Bethesda, MD, United States; ^3^Laboratory of Molecular and Developmental Biology, National Eye Institute, Bethesda, MD, United States; ^4^Laboratory of Immunology, National Institute of Allergy and Infectious Diseases, National Institutes of Health, Bethesda, MD, United States

**Keywords:** Th17, transcriptional regulation, T helper, cytokine, Th1*, CD4 differentiation

## Abstract

While T helper (Th) cells play a crucial role in host defense, an imbalance in Th effector subsets due to dysregulation in their differentiation and expansion contribute to inflammatory disorders. Here, we show that Casz1, whose function is previously unknown in CD4^+^ T cells, coordinates Th differentiation *in vitro* and *in vivo*. Casz1 deficiency in CD4^+^ T cells lowers susceptibility to experimental autoimmune encephalomyelitis, consistent with the reduced frequency of Th17 cells, despite an increase in Th1 cells in mice. Loss of Casz1 in the context of mucosal *Candida* infection severely impairs Th17 and T_reg_ responses, and lowers the ability of the mice to clear the secondary infection. Importantly, in both the models, absence of Casz1 causes a significant diminution in IFN-γ^+^IL-17A^+^ double-positive inflammatory Th17 cells (Th1* cells) in tissues *in vivo*. Transcriptome analyses of CD4^+^ T cells lacking Casz1 show a signature consistent with defective Th17 differentiation. With regards to Th17 differentiation, Casz1 limits repressive histone marks and enables acquisition of permissive histone marks at Rorc, Il17a, Ahr, and Runx1 loci. Taken together, these data identify Casz1 as a new Th plasticity regulator having important clinical implications for autoimmune inflammation and mucosal immunity.

## Introduction

As T helper (Th) cells play beneficial and detrimental roles depending on the cytokine milieu during immune responses (1–3), it is critical to identify the molecular mechanisms that control their differentiation and homeostasis (4). Th differentiation involves a complex interplay among various transcription factors determining the expression and repression of relevant genes, which lead to stipulated and alternative cellular fates, respectively (5, 6). In addition to direct genetic regulatory mechanisms, Th differentiation is regulated by permissive or repressive modifications of histones at relevant gene loci in the chromatin. Th1 and Th2 cells are classical subsets of CD4^+^ T lymphocytes that play central roles in host defense against intracellular bacteria and parasites, respectively. Excessive responses by these cells lead to inflammation and allergy (7). Th17 cells are instrumental in mucosal host defense against extracellular bacteria and fungi (8–11), but a hyperactive Th17 response results in tissue inflammation and autoimmunity, such as multiple sclerosis (1, 12, 13). Transcription factors, such as RAR-related orphan receptor (ROR)-α and -γt, Ikaros, IRF4, FosL2, Runx1, and signal-transducer and activator of transcription (STAT)3, and cytokines, such as IL-6, IL-21, and IL-23 promote Th17 differentiation(2, 11, 14, 15), while c-Maf, STAT5, IL-2, and IL-27 restrain Th17 responses (16–19). They also determine the stabilization of committed cytokine production versus plasticity between Th subsets. Local cytokine cues in tissues and lymphoid organs selectively expand activated T cells of multiple T cell fates, or convert those to cells of another fate, thus generating intraclonal heterogeneity. For example, Th cells which produce both IL-17 and IFN-γ, known as Th1* cells or inflammatory Th17 (iTh17) cells infiltrate in the CNS and contribute to disease pathogenesis during experimental autoimmune encephalomyelitis (EAE) (20). Also, *Candida albicans*–specific Th17 clones can convert to IFN-γ^+^IL-17A^+^ Th1* cells or iTh17 cells (21, 22). A better understanding of the molecular mechanisms instructing commitment versus plasticity in Th subsets in inflamed tissues will provide new therapeutic opportunities and insights for treating pathologies associated with Th responses (7).

Casz1 is an evolutionarily conserved zinc-finger transcription factor originally characterized in *Drosophila*, and required for reprogramming gene expression during neuronal and retinal cell differentiation (23–25). Casz1 is critical for the development of vasculature and differentiation of cardiomyocytes in *Xenopus* and mice (26, 27), and human *CASZ1* locus has been linked to high blood pressure by genome-wide association studies (28). *Casz1* is also a tumor-suppressor gene mapping to chromosome 1p36.22 in humans, which is deleted in neuroblastoma and other cancers (24, 29–32). However, the function of this protein in immune system is unknown. Our preliminary screening experiments showed that Casz1 mRNA is expressed in T cells, and the expression is differentially regulated in different Th subsets. These data rationalized our objective to examine the function of Casz1 in CD4 T cells. Here, we provide evidence that Casz1 regulates the Th17/Th1/regulatory cell differentiation program, at least in part by inducing Th17 signature genes and repressing Th1 signature genes *in vitro*. Casz1 also promotes EAE development as well as oral candidiasis infection recall responses that are Th17 cell-dependent *in vivo*. Loss of inflammation and recall responses in Casz1 deficient mice coincided with reduction in iTh17 (IFN-γ^+^IL-17A^+^) cells in tissues *in vivo*. Collectively, our findings identify Casz1 as a novel cell lineage-determining transcription factor and a potential therapeutic target for diseases involving Th cell-mediated responses.

## Materials and Methods

### Mice and Human Cells

Experiments were performed at Case Western Reserve University under approved protocols in compliance with the Institutional Animal Care and Use Committee’s guidelines. 6- to 10-week-old C57BL/6 and Foxp3^GFP^ mice were purchased from Jackson laboratories and animals of both the genders were used. CD4 conditional *Casz1* deleted mice (CD4-cre Casz1^fl/fl^) were generated as described in Supplementary Experimental Procedures in Supplementary Material. The CD4-Cre transgenic mice were purchased from Taconic Biosciences, Inc. (Taconic NIAID Exchange 4196). C57BL/6 mice were used for back-crossing Casz1-F1 litters for at least 12 generations. Casz1^+/+^(WT) or Casz1^+/−^[Heterozygous (Ht)] littermate mice were used as controls for Casz1 knockout mice. Some replicate experiments, including EAE studies were done at NIAID, NIH under an approved protocol, and in compliance with the NIAID Institutional Animal Care and Use Committee’s guidelines. Human cells were obtained from commercially available PBMC (AllCells).

### Reagents and Antibodies

Purified or fluorochrome conjugated α-CD3 (145-2C11), α-CD28, α-CD25 (3C7), CD4, CD25, IL-2, IL-4, IFN-γ, IL-17F, IL-17A, IL-22, TNF-α, Foxp3, CD45, CD4, CD8, CD11C, and CD19 antibodies were all purchased from eBiosciences (San Diego, CA, USA). Easysep CD4 isolation kits, and PE, biotin, and APC selection kits were purchased from Stemcell technologies (Vancouver, BC, Canada). Recombinant IL-23, IFN-γ, and IL-17A enzyme linked immunosorbent assay (ELISA) antibodies were purchased from eBiosciences. Recombinant IL-6, IL-1β, IL-12, IL-4, and IL-7 were purchased from (BioBasic Inc., Amherst, NY, USA). Human TGF-β1 was purchased from R&D systems. Mouse cells were cultured in complete RPMI-1640 (Hyclone) supplemented with 10% FCS, 100 U/ml penicillin, 100 µg/ml streptomycin, 2 mM glutamine, 10 mM HEPES, 1 mM sodium pyruvate, and 50 µM β-mercaptoethanol.

### Th Differentiation

All *in vitro* experiments using activated or polarized T cells were performed using CD4 T cells pooled from spleen (SPLN) and lymph nodes (LN) of 5–10 mice. CD4^+^ CD44^low^ CD25^−^ naïve T cells (1 × 10^5^) were stimulated in U-bottom 96-well plates using 1 µg/ml of plate-bound α-CD3 and 2 µg/ml α-CD28 antibodies under different Th polarizing conditions for 3–6 days. To rule out T_reg_ contamination, we performed a staining on sorted naïve cells on d0, which showed that more than 99% of the cells were Foxp3 negative. For non-polarizing conditions, CD4^+^ naïve cells were stimulated only with α-CD3 and α-CD28 antibodies with no added cytokines. Naïve cells were polarized in Th1 conditioning milieu with recombinant mouse IL-12 (20 ng/ml) and α-IL-4 (5 µg/ml), Th2 milieu using α-IL-12 (5 µg/ml) and IL-4 (25 ng/ml), iT_reg_ milieu using TGF-β and IL-2, and Th17 milieu using IL-6 (25 ng/ml), IL-1β (20 ng/ml), TGF-β (2 ng/ml), α-IFN-γ (5 µg/ml), and α-IL-4 (5 µg/ml). For sub-optimal/partial Th17 polarization, α-IFN-γ and α-IL-4 antibodies were not added. Where indicated, CD90^+^ T cell depleted splenocytes were added as antigen presenting cells (APC), at a T cell: APC ratio of 10:1 during the initiation of Th1, Th2, and Th17 cultures. APCs were not added for iT_reg_ differentiation. In some experiments, naïve CD4^+^ T cells were carboxy-fluorescein-succinimidyl-ester (CFSE) labeled to assess their proliferation. To inhibit chromatin histone modifications, we stimulated the Ht (CD4-cre Casz1^wt/fl^) and Casz1 deficient naïve cells under Th17 conditions in the presence of dimethyl sulfoxide, 3-deazaneplanocin-A [DZNep; 1 µM; enhancer of zeste 2 (EZH2) inhibitor], GSKS343 (5 µM; EZH2 inhibitor), Trichostatin A (TSA; 100 nM; HDAC inhibitor), and a short chain fatty acid (SCFA) butyrate (100 µM; HDAC inhibitor) that were added 30 min before the initiation of Th17 cultures.

### q-RT PCR Analyses

For q-RT PCR analyses of ROR-γt, Foxp3 IL-17A mRNA, naïve CD4^+^ T cells were stimulated in Th17 cultures as above and RNA was recovered using an RNA isolation Kit (BioBasic). When indicated, CD4^+^ cells were separated from APC using CD90 magnetic beads to determine mRNA levels specifically in CD4^+^ T cells. DNase (Ambion) was used to remove genomic DNA from purified RNA. cDNA was synthesized from total RNA using Mu-MLV reverse transcriptase and oligo-dT primers (BioBasic), and was amplified with SYBR Green PCR Kit (BioBasic) in a real-time PCR machine (Biorad). All primers for PCR (BioBasic) were designed to amplify a coding region within a single exon. The relative amount of cDNA of interest was estimated from its Ct value plotted on a standard curve acquired from the Ct values of a diluted series of DNA. These quantified amounts were normalized to the amount of β-actin mRNA, assigning values of ‘1’ to unstimulated or naïve CD4^+^ T cells that were used as control samples.

### RNA Sequencing (RNA-seq)

Sample preparation, sequencing, and alignment: strand-specific whole transcriptome sequencing libraries were prepared using TruSeq^®^ Stranded Total RNA LT Library Prep Kit (Illumina, San Diego, CA, USA) by following the manufacturer’s procedure. This protocol involved the removal of ribosomal RNA (rRNA) using biotinylated, target-specific oligos combined with Ribo-Zero rRNA removal beads. The RNA was fragmented into small pieces and the cleaved RNA fragments were reverse-transcribed to generate single-strand cDNA using reverse transcriptase and random primers, followed by double-strand cDNA synthesis using DNA polymerase I and RNase H. The resulting double-strand cDNA was used as the input to a standard Illumina library preparation with end-repair, indexed adapter ligation, and PCR amplification to generate sequencer-ready libraries. Six indexed RNA-seq libraries were sequenced on a HiSeq2500 with Illumina TruSeq V4 chemistry (Illumina, San Diego, CA, USA). The FASTQ files with 125 bp paired-end reads were processed using Trimmomatic (version 0.30) to remove adaptor sequences. The trimmed FASTQ data were aligned to the mouse genome (GRCm38) with STAR (version 2.4.2a), which used GENCODE gtf file version 4 (Ensembl 78). STAR software also generated the strand-specific gene read counts. About 75% of 60 million reads per sample were uniquely mapped to the mouse genome for a total of 85% mapping rate. Differential expression analysis: the gene reads count data from Casz1 knockout and wild type control samples, each derived from three independent experiments were normalized with R Package limma (version 3.26.8), and analyzed with unpaired *t*-test. The normalized reads count data were used to generate *z*-scores for heatmap display. Pathway analysis and heat maps: the twofold differentially expressed gene list (Casz1 knockout versus wild type) was analyzed using QIAGEN’s Ingenuity^®^ Pathway Analysis (IPA^®^, QIAGEN Redwood City, www.qiagen.com/ingenuity, IPA Fall Release, September 2015) to generate unbiased molecular and cellular functional analyses. Gene set enrichment analysis (GSEA) was performed using the GSEA software obtained from the Broad Institute (http://www.broad.mit.edu/GSEA). The whole gene list (Casz1 knockout versus wild type) was pre-ranked based on *T*-value before uploading to the GSEA software for pathway analysis. Heatmaps for different cytokine signatures were created in R using the heatmap.2 function in g plots (version 2.17.0).

### Chromatin Immunoprecipitation (ChIP)-qPCR

Chromatin Immunoprecipitation assays were performed using fresh naïve cells on day 4 (d4) *in vitro* activated Th17 cells. In brief, after fixation in 1% formaldehyde, T cells were lysed for 10 min on ice. Chromatin was sheared by sonication in Branson 2510 digital sonifier. Lysates equivalent to 10^7^ cells were used *per* immunoprecipitation. After pre-clearing with protein-A agarose beads (Life technologies), cell lysates were immunoprecipitated overnight at 4°C with 2 µl of α-H3K9me3, α-H3K27me3, α-H3K4me3, α-H4K12Ac, or control IgG antibodies (cell signaling). After washing and elution, crosslinks were reversed for 4 h at 65°C. Eluted DNA was purified and samples were analyzed by qPCR. Data were normalized to input values and expressed as “% input.” Information on primers is provided in the supplementary experimental procedures in Supplementary Material.

### Histology and Intracellular Staining of Cytokines and Proteins

For immunocytochemical periodic acid schiffs (PAS) and hematoxylin and eosin staining, tongue tissues were rinsed with PBS, fixed with 10% formalin overnight, and rehydrated in 70% ethanol. This was followed by paraffin sectioning and staining of paraffin sections (Histoserv, Inc., MD). For single-cell staining, cells were cultured as above, washed in PBS, fixed with Foxp3 fix-perm set (eBioSciences). For intracellular cytokine staining, cultures were re-stimulated with PMA (50 ng/ml) and ionomycin (500 ng/ml) for 4 h, with brefeldin-A (10 µg/ml) added in last 2 h. For pSTAT3 and pSTAT5 staining, the cells were washed, fixed, and were stained with Phosflow staining kit from BD Biosciences using manufacturer’s protocol.

### Flow Cytometry

Data was acquired using BD Fortessa cytometers and were analyzed using FlowJo 9.8 software.

### *In Vivo* Experiments

#### EAE Induction

Experimental autoimmune encephalomyelitis experiments were performed using the myelin oligodendrocyte glycoprotein (MOG_35–55_)/Complete Freund’s Adjuvant (CFA) and pertussis toxin kit (#EK0115) from Hooke’s laboratories (33). Age and sex matched WT and knockout mice, at least 5 per group were EAE induced according to the Hooke’s laboratory protocol. EAE score criteria are provided in the supplementary experimental procedures in Supplementary Material.

#### Oral *Candida* Infection in Mice

Mice were infected as previously described (34, 35). Briefly, they were sublingually infected under anesthesia by placing a 3 mm diameter cotton ball saturated with 0.5 × 10^8^ *Candida albicans* (SC-5314) blastospores for 90 min. Mice were re-infected on d21 after primary infection for assessing the secondary immune responses *in vivo* (36).

### Statistical Analyses

*P*-values were calculated by Student’s *t*-test in Microsoft Excel software using unpaired data, two-way ANOVA test, or Mann–Whitney test in Prism 6.1 (GraphPad Software, Inc.).

## Results

### Casz1 Is Expressed in Mouse and Human CD4^+^ T Lymphocytes

**Figure 1:**
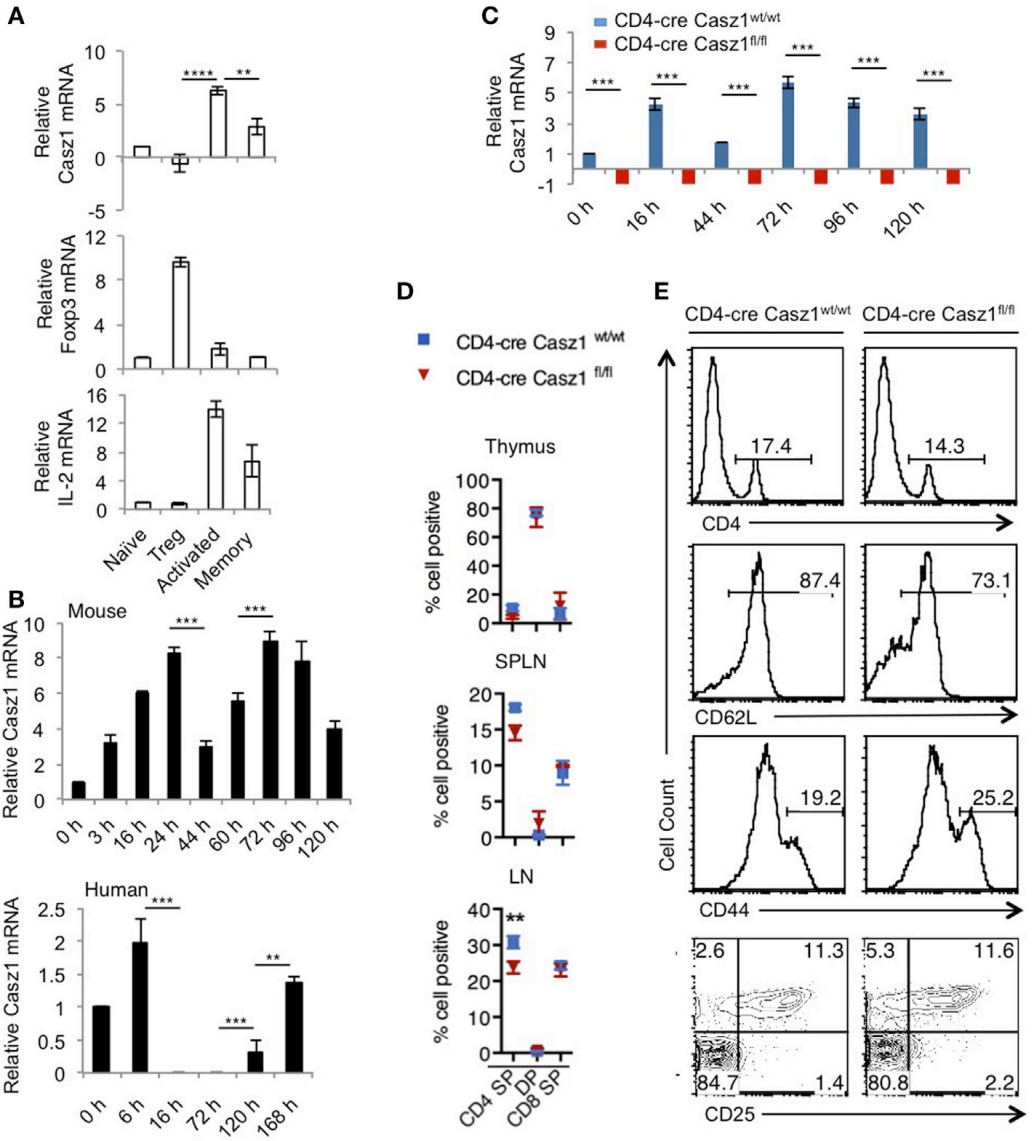
Casz1 mRNA expression in CD4^+^ T lymphocytes. RNA was isolated from fluorescence-activated cell sorting (FACS) sorted CD4^+^CD25^−^GFP^−^CD44^low^ (Naïve) cells, CD4^+^CD25^+^GFP^+^ (regulatory T cells, T_regs_), CD4^+^CD44^high^ (effector/memory) cells pooled from spleen (SPLN) and lymph nodes (LN) of six Foxp3^GFP^ mice and used for Quantitative PCR **(A)** Naïve cells were stimulated with 1 µg/ml of plate-bound α-CD3 and 2 µg/ml of soluble α-CD28 antibodies for 3 days (activated) **(A)**, or for indicated times [**(B)**, upper panel]. Human FACS sorted CD4^+^CD25^−^ CD44^low^ CD45RA naïve cells were activated for indicated times [**(B)**, lower panel]. Phenotype of CD4 conditional Casz1 knockout mice **(C–E)**. **(C)** Naïve CD4^+^ cells from WT (CD4-cre Casz1^wt/wt^) (blue), or knockout (CD4-cre Casz1^fl/fl^) (red) mice were stimulated for indicated times. **(D)** Single cell suspensions from thymus, SPLN, and LN from WT (blue) or knockout (red) mice **(D)**, or SPLN and LN pooled **(E)**, were used for flow cytometry staining in *ex vivo* [Gated on CD4^+^ T cells in second–fourth panels in **(E)**]. Two-way ANOVA multiple comparison tests **(A,B)**, Student’s *t*-tests **(C)**, Two-way ANOVA multiple comparisons test **(D)** were performed to determine the significance. Data from one of three independent experiments are shown.

We first confirmed the expression of Casz1 in various immune cells and found that Casz1 expression was much higher in CD4^+^ T cells than other immune cells (Figure S1 in Supplementary Material). These results prompted us to investigate the function of Casz1 in CD4^+^ T cells. We then determined Casz1 mRNA expression in subsets of CD4^+^ T cells using purified CD4^+^CD25^−^GFP^−^CD44^low^ (Naïve), CD4^+^CD25^+^GFP^+^regulatory T cells (T_regs_), and CD4^+^GFP^−^CD44^high^ (effector/memory) from Foxp3^GFP^ mice by fluorescence-activated cell sorting (Figure S2 in Supplementary Material). We also stimulated naïve cells with 1 µg/ml of plate-bound α-CD3 and 2 µg/ml of soluble α-CD28 antibodies for 3 days to determine its expression in *in vitro* activated CD4^+^ T cells. Quantitative PCR (RT-qPCR) indicated that while naïve CD4^+^ cells and T_regs_ expressed relatively low levels of Casz1 mRNA, activated and memory CD4^+^ T cells expressed threefold to fivefold higher levels of Casz1 mRNA (Figure 1A). We also examined the mRNA levels of Foxp3 and IL-2 to confirm the phenotype of the cells we used. To determine the Casz1 expression kinetics at different time-points, we activated mouse and human naïve cells using α-CD3/α-CD28 as above. Time kinetic analysis indicated that activation of murine CD4^+^ cells showed biphasic regulation of Casz1 with levels rising fourfold within 3–6 h after activation, declining by 44–70 h, and then increasing fivefold after 72 h and remaining elevated compared to naïve cells for up to 5 days after activation. Activated human CD4^+^ cells showed a similar kinetics of CASZ1 expression (Figure 1B), suggesting that Casz1 might have role during T cell activation. Furthermore, high Casz1 expression in *in vitro* induced Th0, Th1, and Th17 subsets, but reduced Casz1 expression in Th2 cells (Figure S3A in Supplementary Material), led us to investigate the function of Casz1 in these subsets. To this end, we generated a mouse line with loxP-flanked *Casz1* alleles (Casz1^fl/fl^) (Figure S3B in Supplementary Material), crossed these mice with those expressing Cre recombinase under CD4 promoter (37), and obtained CD4-cre^+^Casz1^fl/fl^ mice with targeted deletion of *Casz1* in the CD4 cell compartment (Figure S3C and Supplementary method in Supplementary Material). We backcrossed these mice with C57BL/6 mice for 12 generations before using the animals for experiments. qRT-PCR confirmed the absence of Casz1 mRNA expression in CD4-cre Casz1^fl/fl^ mice, comparing those cells with naïve and activated CD4^+^ cells from CD4-cre Casz1^wt/wt^ (WT) mice (Figure 1C). While various *in vitro*-stimulated Th subset cells from WT mice showed variable levels of Casz1 mRNA, CD4-cre Casz1^fl/fl^ (Casz1 knockout; Casz1 deficient) cells showed complete absence of Casz1 mRNA (Figure S3A in Supplementary Material). We observed highly selective ablation of Casz1 gene in Casz1 knockout cells, as Casz1 mRNA expression was intact in non-CD4^+^T cell fraction of the SPLN (Figure S3A in Supplementary Material). To examine the T cell development in these mice, we assessed the frequency of CD4 single positive (SP), CD4^+^CD8^+^double positive, and CD8 SP cell populations in thymus, SPLN, and peripheral LN. The frequency of CD4 SP cells was slightly, but significantly lower in Casz1 knockout mice compared to WT mice (Figures 1D,E, top panel; Figure S4A in Supplementary Material). We then characterized the CD4^+^ cells by assessing the frequency of CD62L^high^ naïve, CD44 high effector/memory, and CD25^+^Foxp3^+^ T_regs_ in 6- to 12-week-old mice. CD4^+^ T cells from Casz1 knockout mice harbored slightly lower frequency of CD62L^high^ naïve cells and moderately higher frequencies of CD44^high^ cells (Figure 1E, second and third panels). However, the frequency of T_regs_ was comparable to WT mice (Figure 1E, bottom panel), and the phenotype of Casz1 knockout mice was normal. These results suggested that whereas CD4^+^ T cell-specific Casz1 deletion slightly reduces CD4^+^ T cell frequencies, CD4^+^ cell phenotype is normal in the periphery. However, we found that the frequency of CD44^high^ IL-17A^+^ natural memory Th17 cells was significantly lower in mucosal tissues of Casz1 knockout mice in *ex vivo* (Figure S4B in Supplementary Material, Y axis; Figure S4C in Supplementary Material) (38). We also observed a small, but reproducible increase in CD44^high^ Foxp3^+^ T_reg_ cells (39, 40) in gut and oral tissues of Casz1 knockout mice in *ex vivo* (Figures S4B, X axis, Figure S4C in Supplementary Material). These results prompted us to focus on Casz1-mediated effects on Th differentiation and inflammation *in vivo*, rather than focusing on reduced CD4 cell frequencies in subsequent experiments.

### Casz1 Controls Th Lineage Differentiation Depending on the Cytokine Milieu

**Figure 2:**
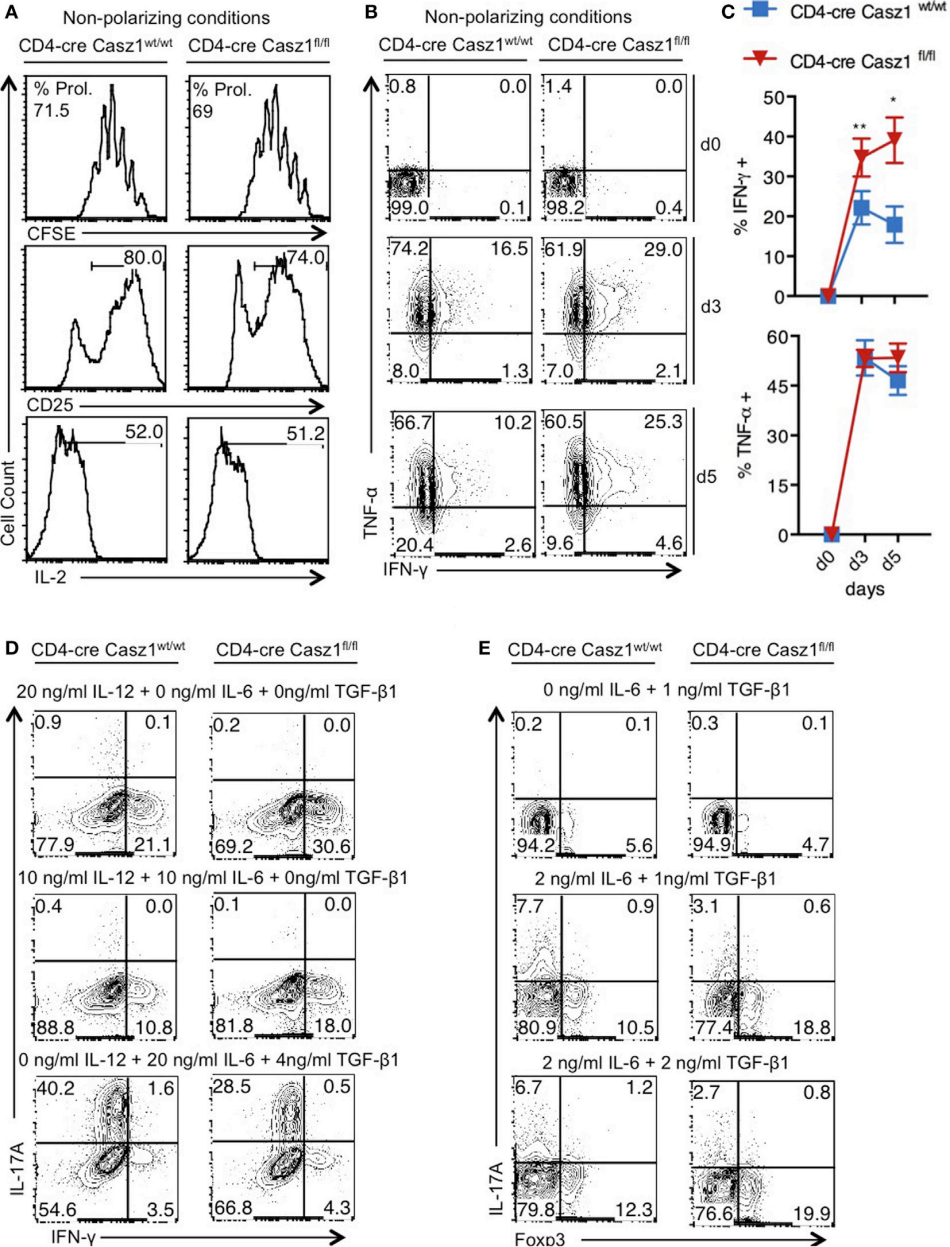
Casz1 controls T helper lineage differentiation depending on the cytokine milieu. **(A–C)** Naïve CD4^+^ cells isolated from WT (left) or knockout (right) mice were stimulated with 1 µg/ml of plate-bound α-CD3 and 2 µg/ml of soluble α-CD28 antibodies for 4 days **(A,B)** or for indicated times and subjected to flow cytometry. The percentage of divided cells is indicated as % Prol. in **(A)**. **(C)** Averages from 3 independent experiments are plotted. *P < 0.05, as determined by unpaired Student’s *t*-test. **(D,E)** Cells were stimulated as in **(A)**, in the presence of indicated cytokines for 4 days before the intracellular staining and flow cytometry. Data represent one of the five independent experiments showing similar results. Gates were drawn using unstain or isotype controls.

First, we sought to determine whether Casz1 affects CD4^+^ T cell activation and proliferation *in vitro*. We sorted CD4^+^CD44^low^CD25^−^ naïve cells from WT and Casz1 knockout mice and stimulated them using 1 µg/ml of plate-bound α-CD3 and 2 µg/ml of soluble α-CD28 antibodies in the absence of polarization conditions, and assessed their proliferation and activation on d4 after stimulation. CFSE dilution assay revealed that proliferation of Casz1 knockout cells was comparable to WT cells (Figure 2A, top panel). Also, the expression of activation markers, such as CD25, CD44, and IL-2, as well as Foxp3 were comparable between CD4^+^ T cells from these mice at various time points after stimulation (Figure 2A, middle and bottom panels; Figure S5 in Supplementary Material). However, we found that IFN-γ secretion was 15–20% higher in Casz1 knockout CD4^+^ T cells, while TNF-α expression did not show significant changes, compared to WT CD4^+^ T cells, as assessed by intracellular staining on d4 after stimulation (Figures 2B,C). Because the Casz1 deficient CD4^+^ T cells defaulted to IFN-γ producing Th1 subset compared to WT cells, we hypothesized that Casz1 may control Th cell specification events. Since, cytokine concentrations dictate the Th differentiation into different subsets, we activated naïve cells with different concentrations of Th specification cytokines, such as IL-12 (Th1), IL-6 (Th17), and TGF-β (iT_reg_), mimicking suboptimal Th polarization conditions. Depending on the concentration of IL-12 and TGF-β, the frequency of IFN-γ and Foxp3 expressing cells was 7–11% higher in Casz1 deficient cultures compared to WT cultures (Figures 2D,E). However, at all concentrations of IL-6 in partial Th17 polarization milieu, IL-17A production was reduced by up to fourfold in Casz1 deficient cultures than WT cultures (Figures 2D,E). Taken together, these data indicate that while Casz1 inhibits IFN-γ and Foxp3 expression depending on the cytokine milieu, it potently promotes the expression of IL-17A, a key component of the Th17 phenotype.

### Casz1 Is a Critical Component of Th17 Lineage Differentiation

**Figure 3:**
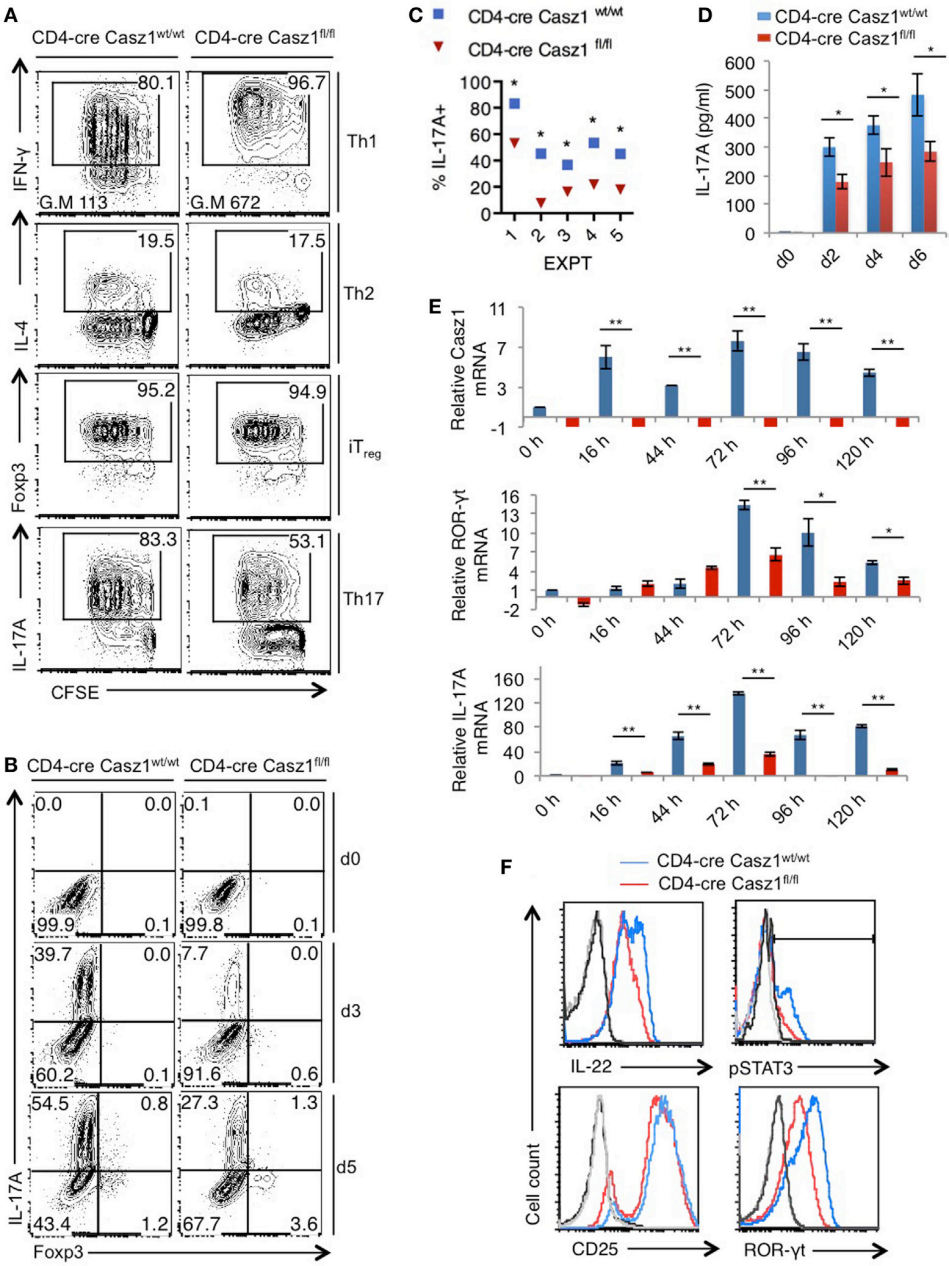
Casz1 promotes IL-17A expression in Th17 cultures. **(A)** Casz1^+/+^WT or Casz1 knockout naïve CD4^+^ T cells were cultured under Th1 (top), Th2 (second panel), iT_reg_ (third panel), or Th17 (bottom panel) conditions for 5 days. Th1, Th2, Th17 cells were stimulated with antigen presenting cells (APC), and iT_regs_ without APC (Figure S6 in Supplementary Material). Intracellular cytokine staining was performed in PMA/ionomycin re-stimulated cells. Data from flow cytometric analyses (gated on CD4^+^ cells) showing the proliferation (carboxy-fluorescein-succinimidyl-ester, X axis), percentage of IFN-γ (top), IL-4 (second panel), Foxp3 (third panel), and IL-17A (bottom panel) positive cells (Y-axis). Gates were drawn based on unstained and un-stimulated controls (not shown). G.M of IFN-γ staining in Th1 cells (top panel) is indicated at the bottom of the plots. **(B)** Casz1^+/+^WT or Casz1 knockout naïve CD4^+^ cells were stimulated under Th17 skewing conditions in the presence of APC for indicated days before flow cytometry. **(C)** Casz1^+/+^WT (blue) or Casz1 knockout (red) naïve CD4^+^ cells were stimulated under Th17 conditions as in (**B**) in five independent experiments, and the frequency of IL-17A^+^ cells was determined by flow cytometry on day 4 (*P < 0.05 was determined by Wilcoxon matched-pairs signed rank test). **(D)** Supernatants were collected for enzyme linked immunosorbent assay at indicated time-points. **(E)** Casz1^+/+^WT (blue) or Casz1 knockout (red) naïve CD4^+^ cells were stimulated under Th17 conditions for indicated time-points as in **(B)**, and APCs were removed by MACS before determining the mRNA levels of Casz1 (top), RAR-related orphan receptor-γt (middle), and IL-17A (bottom) in CD4^+^ T cells by qPCR. **(F)** Casz1^+/+^WT (blue) or Casz1 knockout (red) cells were stimulated as in **(B)** for 3 days and were stained for indicated proteins (gated on CD4^+^ cells). For phosphorylated STAT-3 (pSTAT3) and pSTAT5 staining, cells were washed and re-stimulated with IL-6 or IL-2, respectively for 30 min before fixation. The results are representative of at least three independent experiments (*P < 0.05, as determined by unpaired Student’s *t*-test or Mann–Whitney test). Gates were drawn using unstain or isotype controls.

**Figure 4:**
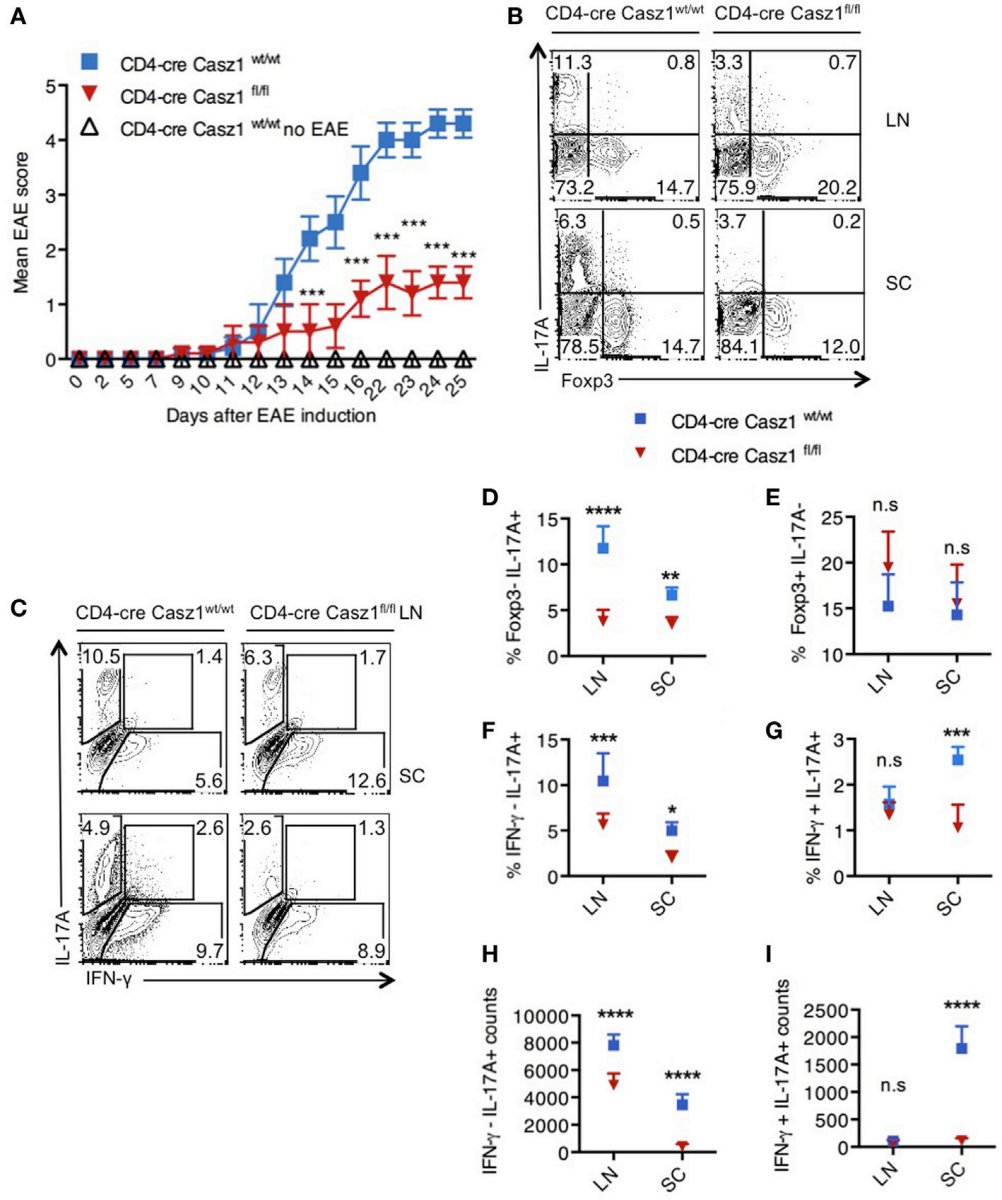
Loss of Casz1 ameliorates Th17 inflammation during experimental autoimmune encephalomyelitis (EAE) *in vivo*. **(A)** EAE was induced in 8- to 10-week-old Casz1^+/+^WT (blue; *n* = 7) or Casz1 knockout (red; *n* = 6) mice using myelin oligodendrocyte glycoprotein (MOG) peptide. Some Casz1^+/+^ WT control mice were left untreated (white; *n* = 6; No EAE). EAE scores were recorded in masked fashion. Mean scores ± SEM are plotted. ***P = 0.002 was determined by two-way ANOVA *t*-test. **(B)** On day 10 after EAE induction, pooled axillary/inguinal lymph nodes (LN), or spinal cords (SC) were harvested for re-stimulation with MOG peptide and intracellular staining of Foxp3 (X axis) and IL-17A (Y axis) **(B)**, or IFN- γ (X axis) and IL-17A (Y axis) **(C)** (Gated on CD4^+^ cells). **(D–I)** The frequencies and cell counts of cytokine and Foxp3 expressing cells in LN or SC are plotted (gated on CD4^+^ cells). Mean ± SEM of each group is shown. Two-way ANOVA with Bonferroni’s *post hoc* analyses were performed to determine significance. These data represent three independent experiments. Gates were drawn using unstain or isotype controls.

Because Casz1 controls genes instructing Th1/Th17 specification program in partial polarization conditions, we next sought to investigate the role of Casz1 in synchronized Th cells that are activated under complete polarization conditions (see Methods) (Figure S6 in Supplementary Material). We activated the naïve cells under Th1 (IL-12, α-IL-4, and IL-2), Th2 (α-IL-12, IL-4, and IL-2), iT_reg_ (TGF-β and IL-2), and Th17 (α-IFN-γ, α-IL-4, IL-6, TGF-β, and IL-1β) polarization conditions in the presence of α-CD3, α-CD28, and APC. After a 5-day stimulation, there were no differences in proliferation between WT and Casz1 knockout in any of the Th cell-type cultures, as assessed by CFSE dilution (Figure 3A, X axis). Consistent with the results from partial polarization conditions, the proportion of IFN-γ producers was 16% higher in Casz1 knockout Th1 cultures than Casz1^+/+^WT Th1 cells under complete Th1 polarization conditions (Figure 3A, top panel). We also found a higher geometric mean fluorescence intensity of IFN-γ staining in Casz1 knockout cultures (Figure 3A, top panel). However, there were no differences between Casz1 knockout cultures and WT cells in IL-4 and Foxp3 expression in Th2 and iT_reg_ conditions, respectively (Figure 3A, second and third panels). However, under Th17 conditions, IL-17A production was significantly and consistently lower in Casz1 deficient cultures compared to WT controls at all time-points assessed after stimulation (Figure 3A, bottom panel, Figure 3B, Y-axis, Figure 4C). No significant differences could be detected in Foxp3 expression between WT and knockout cells in Th17 cultures (Figure 3B, X-axis). ELISA also showed that the supernatant from Casz1 deficient cultures had 1.5- to 2-fold lower IL-17A protein levels than WT controls (Figure 3D). These striking differences in Th17 phenotype between WT and Casz1 knockout T cells led us to examine polarized Th17 cells in more detail. qRT-PCR confirmed the absence of *Casz1* mRNA and ~two to threefold decrease in *Rorc* and *Il17A* mRNA levels in Casz1 deficient T cells as compared to WT cells isolated from the cultures at various time-points (Figure 3E). Most of the IL-17A^+^ cells co-expressed IL-17F, and the frequency of IL-17A^+^IL-17F^+^ double producers was also diminished by 40% in Casz1 deficient cells (Figure S7 in Supplementary Material). Also, the levels of other Th17-associated proteins, such as IL-22, ROR-γt, and phosphorylated STAT-3 (pSTAT-3) were much lower in Casz1 deficient cells than WT Th17 cells (Figure 3F). However, Casz1 deficient cells did not show a major defect in CD25 upregulation, or default to IFN-γ producing Th1 lineage or iT_reg_ lineage under the influence of strong Th17 differentiation signals *in vitro* (Figure 3B, X-axis; Figure S8 in Supplementary Material). We found similar results when we compared Casz1^+/+^Ht and Casz1 deficient Th17 cells, Ht and WT mice showed similar phenotype (see Figure 7). Taken together, while Casz1 is not essential for activation and proliferation of CD4^+^ T cells, it appears to be required for Th17 differentiation.

### Casz1 Deficiency Lowers EAE Severity Correlating to IL-17A Reduction

The above results indicated that Casz1 might control inflammation in a Th17-dependent EAE disease model, which mimics the demyelinating disease pathology in multiple sclerosis. To determine the function of Casz1 in EAE progression, we induced the disease in WT (*n* = 5) and Casz1 knockout mice (*n* = 6) (33). Casz1 knockout mice exhibited significantly attenuated disease severity, with lower EAE scores compared to WT mice (Figure 4A). On days 10 or 15 after EAE induction, we isolated single cell suspensions of CD4^+^ cells from axillary and inguinal LN and spinal cords (SC) to assess cytokine production. Flow cytometry analyses revealed that CD4^+^ T cells in Casz1 knockout mice contained significantly lower IL-17A producing T cells compared to WT mice (Figures 4A,C, Y-axes, Figure 4D). However, the overall CD4^+^ T cell numbers, the expression of GMCSF and CCR6 in CD4^+^ T cells were unchanged *in vivo* (data not shown). Consistent with our *in vitro* observations in partially polarized cultures (Figures 2D,E), the decrease in IL-17A producing cells coincided with the increase in Foxp3^+^ cells, as well as IFN-γ secreting cells in Casz1 knockout mice (Figures 4B,C, X-axes, Figures 4E,F). However, the frequency of IL-17A^+^ IFN-γ^+^ cytokine producers was substantially lower in Casz1 knockout than WT mice (Figure 4G). These results show that Casz1 deficiency in T cell lineage results in a diminished iTh17 response and a failure to develop EAE.

### Casz1 Deficient Mice Display Impaired Recall Response to *Candida albicans* Challenge, Coinciding with Lower IL-17A Production in CD4^+^ T Cells *In Vivo*

**Figure 5:**
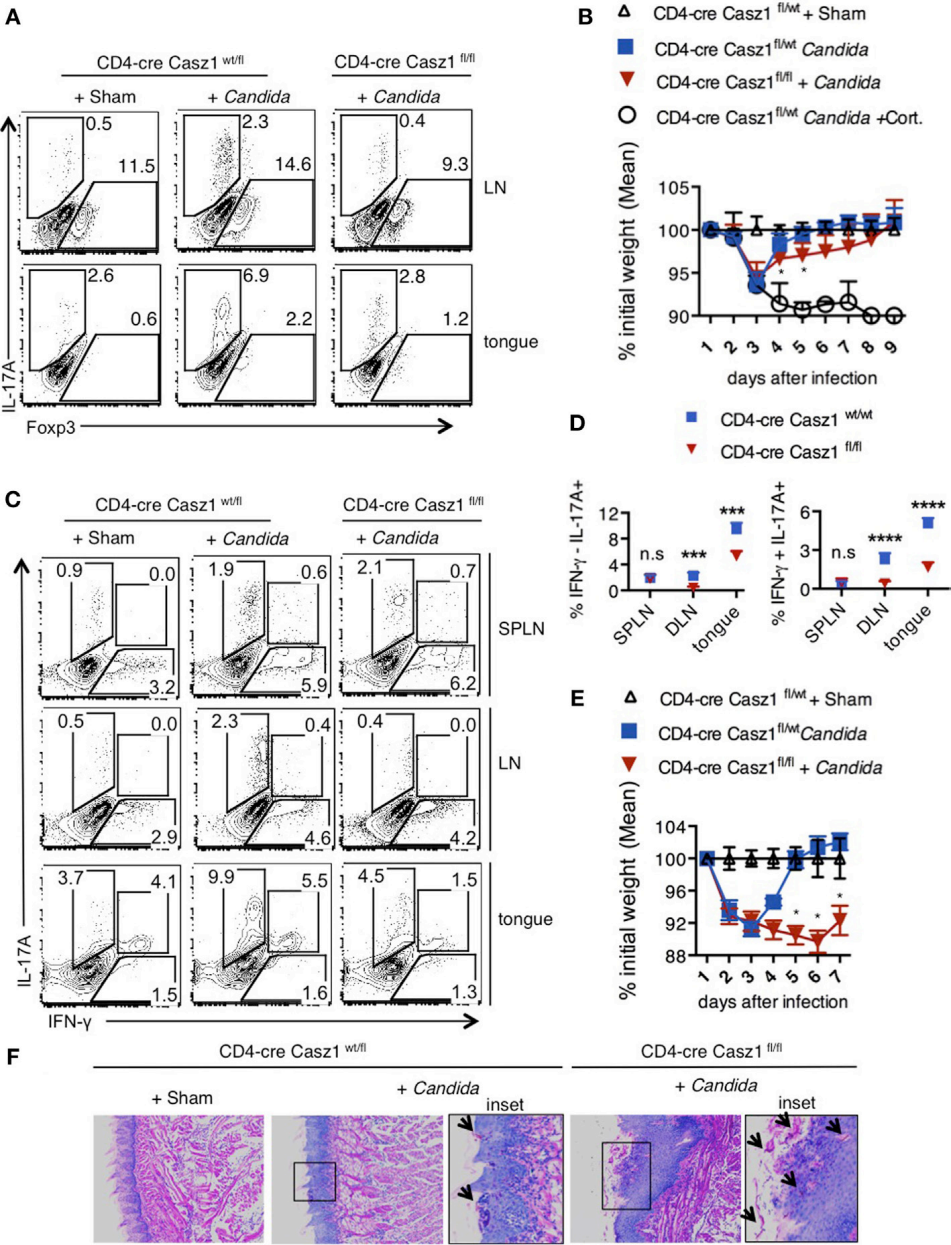
Loss of Casz1 impairs IL-17A production and Th17 recall responses during *Candida* infection in mice. **(A)** 8- to 10-week-old CD4-cre Casz1^wt/fl^ (*n* = 5) or CD4-cre Casz1^fl/fl^ (*n* = 5) were infected with *Candida* as described in Methods. Some control mice received PBS (Sham; *n* = 5) or immunosuppressed with Cortisone injection (Cort; *n* = 3). Pooled axillary and cervical draining lymph nodes (LN) and the tongues were harvested and restimulated with PMA/Ionomycin on day 3 after primary infection for IFN-γ (X-axis) and IL-17A (Y-axis) staining (gated on CD4^+^ cells). Data on day 5 showed similar results (not shown). **(B)** Percent weight change on indicated days after primary infection. **(C,D)** Mice were infected as in **(A)**, and re-infected on d21 after primary infection. Two days after reinfection, spleen (SPLN), draining LN, and tongues were processed for flow cytometry. **(E)** Percent weight change on indicated days after re-infection. **(F)** On day 5 after reinfection, histology sections of the tongue were stained with periodic acid schiffs to detect the fungus. Microscopic images of the slides that were viewed at 200× magnification, with insets showing further magnification [arrows show *Candida* (pink)]. At least five independent experiments, comparing heterozygous with knockout animals, as well as WT with knockout animals showed similar results. Two-way ANOVA test was performed to determine the significance. Gates were drawn using unstain or isotype controls.

We next examined the role of Casz1 during oral *Candida* infection, a model in which IL-17A and Th17 cells are required for anti-fungal defense in the host (35, 41). Fungal lesions and inflammation in the tongue, decreased food intake, body weight loss, and the eventual moribund state are characteristics of susceptibility of the mice to oropharyngeal candidiasis (OPC) infection. As Casz1^wt/fl^ Ht and WT mice showed similar phenotype in our *in vitro* experiments, we used Ht mice as controls in this experiment (see above, and Figure 7). We orally infected CD4-cre Casz1^wt/fl^ Ht control (*n* = 5) and Casz1 knockout (*n* = 5) mice with *Candida* or PBS sham control as described previously (41). One group of mice also received cortisone as an immunosuppressant (*n* = 3). We quantified IL-17A cytokine levels in pooled axillary and cervical draining LN and tongues from the 3 or 5-day infected mice (Figure 5A). Casz1 knockout mice exhibited a significantly lower frequency of IL-17A producers and Foxp3^+^ cells among CD4^+^ cells, but no significant difference in the percentage of IFN-γ producing cells, compared to controls (Figure 5A). Interestingly, there was a slight, but significant delay in recovery from infection-dependent body weight loss in Casz1 knockout mice, albeit both the control and Casz1 knockout groups eventually recovered (Figure 5B). As expected, cortisone group mice progressively lost weight. There were no significant differences in fungal burden between the control and knockout groups, when assessed on day 7 after infection (Figure S9A in Supplementary Material). Because Th17 cells are predominantly involved in recall responses during OPC (36), we next investigated the role of Casz1 during recall responses to *Candida*. Analysis of SPLN, LN, and tongue infiltrates at 2 days after secondary infection revealed that the frequency of IL17A producing CD4^+^ T cells was significantly reduced in Casz1 knockout mice (Figures 5C,D). Also, absence of Casz1 caused a significant diminution in IFN-γ^+^IL-17A^+^ iTh17 cells (Th1*) in draining LN and tongue during secondary infection (Figure 5C). While the frequency of Foxp3^+^ T_reg_ cells decreased in Casz1 knockout groups during primary infection, there was no significant difference in Foxp3 expression between groups during secondary infection (Figure 5A; Figure S9B in Supplementary Material, X-axis). Strikingly, coinciding with a lower Th17 recall response in Casz1 deficient group, we observed a pronounced body weight loss (Figure 5E), abundance of fungal hyphae in the tongue (Figure 5F; Figure S9C in Supplementary Material, left) and heightened tongue inflammation indicated by increased immune cell infiltration (Figure S9C in Supplementary Material, right).

### Casz1 Favors Th17 Specification Program

**Figure 6:**
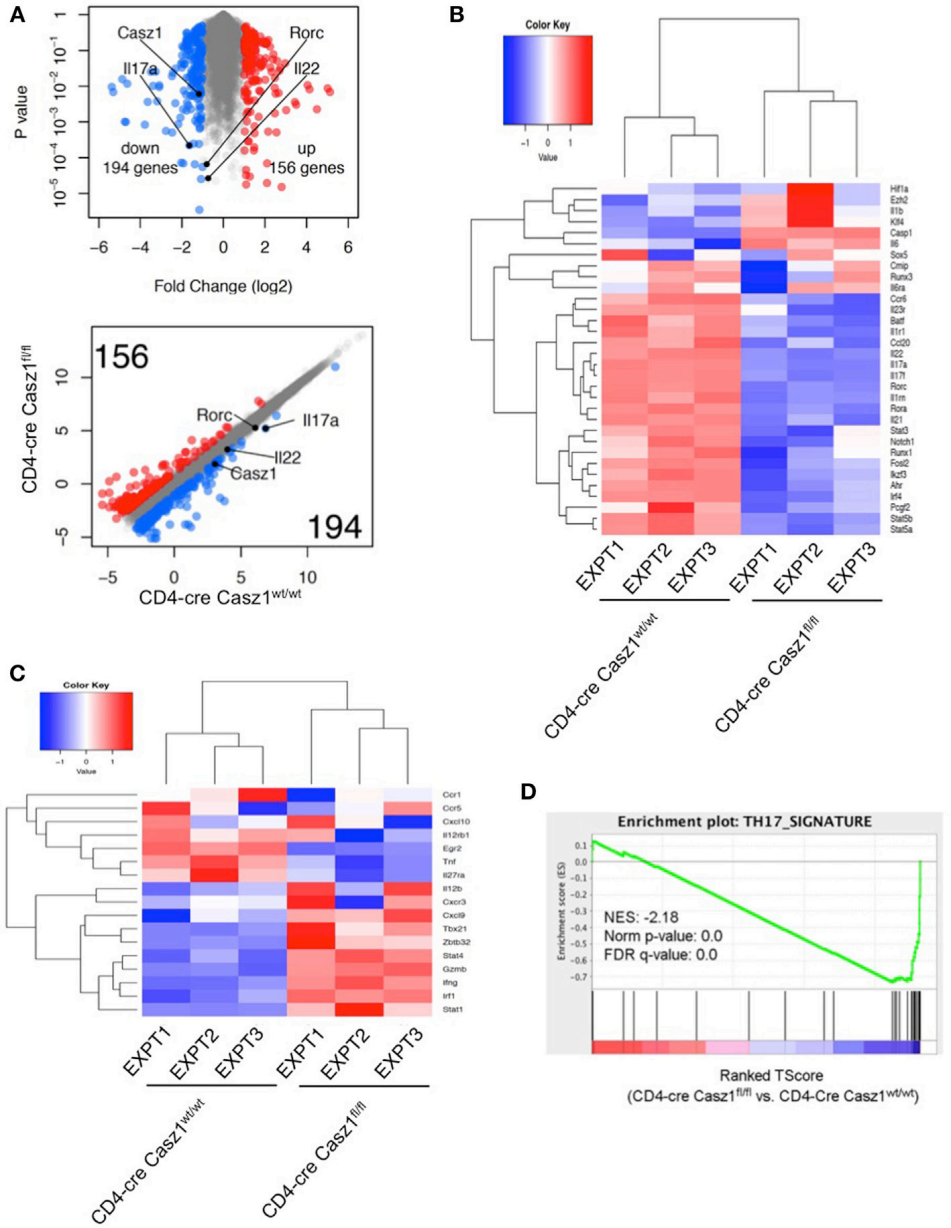
RNA sequencing (RNA-seq) analysis shows that Casz1 favors Th17 specification program. Volcano plot [**(A)**, top], Scatter plot [**(A)**, bottom], and heat maps of genes encoding literature curated Th17 signature proteins **(B)** and Th1 regulatory proteins **(C)**, derived from RNA-seq analysis comparing partially polarized CD4-cre Casz1^fl/fl^ Th17 cells and CD4-cre Casz1^wt/wt^ Th17 cells. Th17 and Th1 signature genes were defined based on the published literature. In **(B,C)**, the upper dendrograms illustrate the results of unsupervised hierarchical clustering of all six samples based on the expression of these Th17 signature genes. **(D)** Gene set enrichment analysis (GSEA) was performed using the whole gene list generated from RNA-seq. This whole gene list was pre-ranked based on *T*-Score, then uploaded to GSEA software. Abbreviations: *Nes*, normalized enrichment score; *Nom*, nominal; *FDR*, false discovery rate. Th17 signature gene list that was used for GSEA is depicted in Figure S10B in Supplementary Material. Data are representative of three independent biological replicates from cells derived from three independent experiments.

To assess whether Casz1 regulates the broad Th program, we performed transcriptome analysis using high-throughput RNA-seq of the WT and Casz1 deficient CD4^+^ T cells stimulated under partial Th17 polarization conditions, *i.e*., without blocking IFN-γ and IL-4 (see Figure S6 in Supplementary Material). We added Th17 cytokines, such as IL-6, TGF-β, and IL-1β, but did not block IFN-γ and IL-4, such that we can obtain an impartial assessment of the effect of Casz1 on the global Th differentiation program while focusing on Th17 program. Transcriptome analysis indicated that 194 genes including Casz1, IL-17A, and IL-22 were upregulated, while 156 genes were downregulated in WT cells compared to Casz1 deficient CD4^+^ T cells (Figure 6A). Global pathway analysis identified alterations in immune cell signaling, inflammatory diseases, cancer, infectious diseases, humoral, and cell-mediated responses (Figure S10A in Supplementary Material). Strikingly, loss of Casz1 led to significant decreases in key Th17 specification genes (6) that included *Rora, Ahr, Runx1, Irf4, Fosl2, Stat3, Ccr6, Il23r, Il1r, Il17a, Il17f, Il22, and Il21* (Figure 6B). We also found that Casz1 restrains the expression of some of the genes that instruct Th1 differentiation program, namely *Tbx1, Irf1, Stat1, Stat4, and Ifng* (Figure 6C). GSEA (42) indicated a significant negative enrichment of literature-curated Th17 signature genes in Casz1 deficient cells (20 of 31 genes) (*p* < 0.001, FDR *q* < 0.001) (Figure 6D; Figure S10B in Supplementary Material). Casz1 also restricts the expression of some of the genes driving Th1 lineage commitment. These data are consistent with a key role for Casz1 as a regulator of Th plasticity program and necessary for robust Th17 differentiation.

### Casz1 Affects the Chromatin State of Th17 Lineage Genes

**Figure 7:**
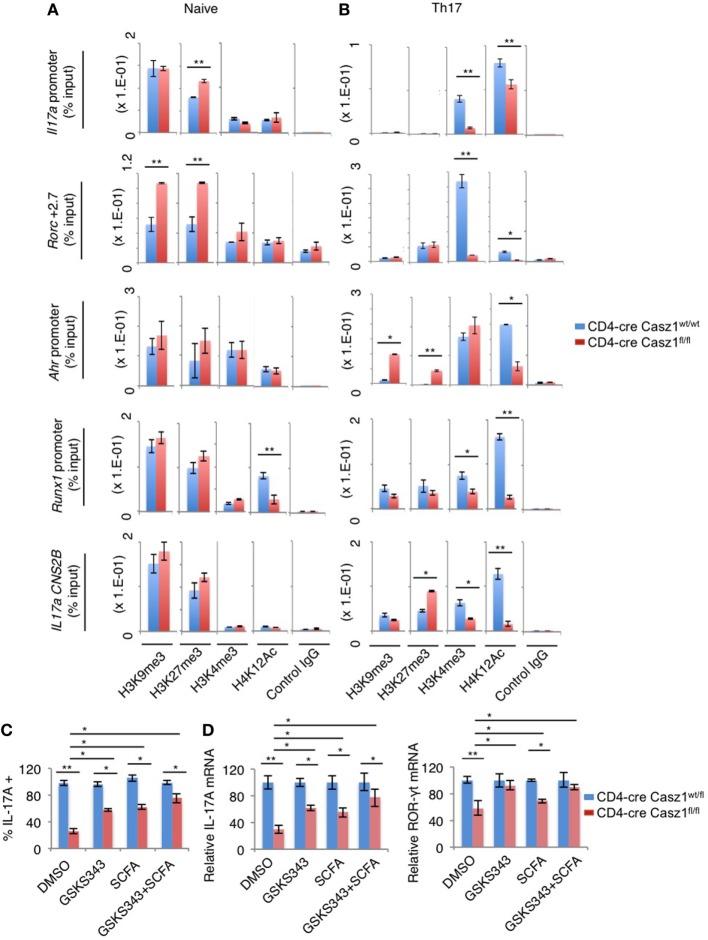
Casz1 promotes the expression of Th17 genes by affecting chromatin modifications in Th17 lineage genes. Casz1^+/−^WT (blue) or Casz1 knockout (red) naïve cells were fixed **(A)**, or stimulated as in Figure 3B for 4–5 days before fixation **(B)**, for chromatin immunoprecipitation using α-H3K9me3, α-H3K27me3, α-H3K4me3, α-H4K12Ac, or control IgG antibodies and qPCR primer pairs for the indicated genes. Casz1^+/−^heterozygous (blue) or Casz1 knockout (red) naïve CD4^+^ cells were stimulated under Th17 conditions as in Figure 3B for 4 days in the presence of dimethyl sulfoxide or the indicated inhibitors. The frequency of IL-17A^+^ cells was determined by flow cytometry (gated on CD4^+^CD44^high^ cells) **(C)** Antigen presenting cells were removed by MACS purification of CD4^+^ T cells before determining the mRNA levels of IL-17A (left) and RAR-related orphan receptor-γt (right) in CD4^+^ T cells by qPCR **(D)**. Results represent 3 independent experiments. *P < 0.05, as determined by unpaired Student’s *t*-test.

Next, we sought to determine the mechanism underlying Casz1-mediated control of Th differentiation program focusing on Th17 signature genes. Casz1 has been shown to interact with histones and recruit nucleosome remodeling and histone deacetylase complex, and Polycomb complex histone methytransferase subunits EZH2 to regulate gene transcription in HEK293T and neuroblastoma cells (43). Also, Casz1 is a zinc-finger protein similar to Ikaros (44), a protein that controls Th differentiation genes through epigenetic modifications (45–48). Casz1 is present in an evolutionarily conserved transcription factor pathway in which Ikaros regulates Casz1 expression in neural progenitors (49). While diminution of ROR-γt and activated STAT-3 may provide some mechanistic insight into reduced IL-17A production in Casz1 deficient Th17 cells, we explored whether Casz1 also mediates some of its effects through epigenetic mechanisms. We compared WT and Casz1 deficient CD4^+^ T cells for chromatin modifications at the promoters and the regulatory regions of the key Th17 genes by ChIP assay. We first evaluated repressive (H3K9me3, H3K27me3) histone modifications at several sites, including the promoter and CNS-2 regions of the *Il17a* gene, +2.7, and +5.0 sites of the *Rorc* gene, and proximal promoter regions of *Ahr* and *Runx1* genes, which are known to contain chromatin modifications associated with Th17 commitment (50, 51). We observed increased amounts of H3K27me3 modifications at *Il17a* promoter and *Rorc* loci in Casz1 deficient naïve cells compared to WT controls (Figure 7A). While H3K9me3 repressive modifications were also present at higher levels at *Rorc* locus, H4K12Ac permissive modifications were lower in non-polarized naïve Casz1 deficient cells compared to WT controls (Figure 7A). However, as expected, cells activated under Th17 polarizing conditions exhibited much lower repressive modifications than naïve cells. Such modifications were still higher in Casz1 deficient cells compared to WT cells, in *IL17a CNS2B* and *Ahr* loci (Figure 7B). Strikingly, in activated Th17 cells, permissive histone marks (H3K4me3, H4K12Ac) increased, but were detected at much lower levels on all the loci examined in Casz1 knockout Th17 cells (Figure 7B). To validate the alterations in histone methylation and acetylation at the Th17 signature gene loci contribute to decreased IL-17A production in Casz1 deficient cells, we treated Th17 cultures with DNA methyl-transferase EZH2 inhibitors and histone deacetylase (HDAC) inhibitors and determined the IL-17A production. We stimulated the Ht (CD4-cre Casz1^wt/fl^) and Casz1 deficient naïve cells under Th17 conditions in the presence of GSKS343 (EZH2 inhibitor) and a SCFA HDAC inhibitor butyrate. GSKS343 and SCFA partially blocked the activation of CD4^+^ T cells, therefore, we gated on CD44^high^ activated cells to determine the intracellular IL-17A expression. GSKS343 and SCFA alone moderately, but significantly increased IL-17A production in Casz1 deficient cells. However, combinations of these inhibitors restored the IL-17A production of Casz1 deficient cells comparable to Ht control levels (Figure 7C). Of note, such treatment of Casz1 deficient cells also normalized their IL-17A and ROR-γt mRNA expression to control levels (Figure 7D). Collectively, the increase in repressive marks and reduction in activating histone marks in Casz1 deficient cells indicate that Casz1 influences the chromatin landscape of Th17 cell lineage-determining genes. These results also established a key requirement of the Casz1-dependent histone modifications in genes encoding several markers of Th17 differentiation in instructing the Th17 program.

## Discussion

In this study, we have identified that Casz1 is expressed in CD4^+^ T lymphocytes and is essential for determining Th1/Th17 balance, depending on the context. By employing CD4-conditional Casz1 knockout mice and global transcriptome analyses of CD4^+^ T cells, we have demonstrated that Casz1 plays a crucial role in Th plasticity, mainly instructing Th17 lineage differentiation program. The physiological requirement of Casz1 in Th17 cell differentiation and disease pathogenesis was demonstrated by *in vivo* experiments showing that Casz1 deficiency leads to amelioration of EAE disease and a compromised recall immunity of the host toward OPC infection (Figures 4 and 5). On one hand, Casz1 plays a detrimental role by promoting iTh17 cell induction among CNS infiltrating cells and exacerbates the initiation and progression of EAE disease. It should be noted that Casz1 is required for EAE disease despite its ability to restrain IFN-γ *in vivo* (Figures 4B,C). On the other hand, Casz1 coordinates a protective Th17 recall responses without altering the frequency of IFN-γ-single producers during a mucosal infection. However, in the both the models, absence of Casz1 causes a significant diminution in IFN-γ^+^IL-17A^+^ iTh17 (Th1*) cells in tissues, correlating to reduced inflammation, and recall responses *in vivo*. Whether Th17 plasticity causes Th1* cell generation in tissues and Casz1 controls this process remains to be studied. Although Casz1 reduces Foxp3 expression when activated with low concentrations of TGF-β *in vitro* (Figure 3B), there were no differences observed between WT and knockout cells under complete iT_reg_ polarization conditions *in vitro* or *in vivo* (Figure 3A). This would not be surprising considering the reciprocal relationship between Th17 and T_reg_ lineages. Moreover, the frequency of CD25^neg^Foxp3^+^ cells was consistently higher in the absence of Casz1 *in vivo*, implying that Casz1 plays a role in regulating these cells. Although CD25^neg^Foxp3^+^ cells are implicated in T_reg_ plasticity, the role of Casz1 in regulation of this population remains unclear. These data highlight the ability of Casz1 to contextually program Th lineage differentiation, depending on the cytokine and immunological location *in vivo* (Figures 4 and 5). When CD4^+^ T cells are activated under Th17 inducing conditions, loss of Casz1 results in dramatic reduction of other Th17 hallmark effector molecules and lineage-determining transcription factors, including IL-17F, IL-21, FosL2, Ikaros, Ahr, Runx1, Rorc, IL1R1, and IL-23R (Figure 6B). Under Th17 and Th1 polarization conditions *in vitro*, Casz1 restricts IFN-γ production (from IFN-γ-single producers) (Figures 2B–D and 3A), as in the EAE model. Consistently, our transcriptome analyses also show that many of the Th1 signature genes are significantly up-regulated in Casz1 deficient CD4^+^ T cells, even under suboptimal Th17 milieu *in vitro* (Figure 6C). Taken together, these results show that while Casz1 favors Th17 lineage *in vitro* and *in vivo*, it may also play a negative regulatory role in Th1 and iT_reg_ lineage commitment depending on the cytokine milieu. The mechanisms underlying Casz1-mediated restriction of T_reg_ and Th1 genes, and induction of iTh17 cells or Th1* cells (IFNγ^+^IL-17A^+^) in tissues remain to be investigated in the future. Consistent with the role of Casz1 in promoting Th17 cells in mucosal tissues, the frequency of memory Th17 cells is also lower in mucosal tissues of Casz1 knockout mice *ex vivo* (Figure S4B in Supplementary Material, Y axis). These results not only corroborate our results demonstrating the positive regulatory role of Casz1 in Th17 differentiation, but also highlight the possibility that Casz1 may be involved in the generation of memory Th17 cells *in vivo* (38), as well as IFNγ^+^IL-17A^+^ (Th1*) cells during memory Th17 responses, which will be studied in the future. Because we have deleted Casz1 in CD4^+^ compartment, some of the effects may arguably be attributed to CD4^+^ macrophages *in vivo*. *In vitro* experiments using purified CD4^+^ T cells that showed Th program alterations in Casz1 knockout cells demonstrates that the observed effects are predominantly driven by CD4^+^ T cell defects *in vivo*. Whether the loss of Casz1 additionally affects natural killer T cells and innate lymphoid cells remains to be investigated in the future. Mechanistically, our data show that Casz1 promotes the Th17 program at least in part by epigenetic regulation of several of the Th17 signature genes. Analysis of histone modification patterns reveals that Casz1 affects chromatin modification in Th17 genes, both in peripheral naïve CD4^+^ cells and in TCR stimulated Th17 cells. On one hand, Casz1 maintains Th17 gene expression potential by inhibiting the attainment of H3K27me3-dependent repressive configuration at the *Il17a* and *Rorc* elements, and decreases H3K9me3 repressive marks at *Rorc* promoter in naïve CD4^+^ T cells (Figure 7A). On the other hand in activated Th17 cells, activation of TCR, IL-6, and TGF-β1 receptor signals overcomes the differences in H3K27me3-dependent repressive states between WT and Casz1 deficient cells. However, in activated Th17 cells, Casz1 autonomously promotes the acquisition of permissive H3K4me3 as well as H3K12Ac modifications in key Th17 genes (Figure 7B). Consistent with these results we found that inhibition of H3K27 tri-methylation by EZH2 inhibition, only partially reversed the defect in IL-17A production by Casz1 deficient cells activated under Th17 conditions. Altering methylation and acetylation of histone modifications alone using chemical inhibitors does not completely restore Th17 defects in Casz1 knockout cells (Figures 7C,D), suggesting additional functions of Casz1 in Th cell differentiation. Recently, Casz1 has been shown to interact with T-box transcription factor (52). Whether such direct interactions between Casz1 and other transcription factors regulate T cell differentiation remains to be seen.

We did not examine whether Casz1 binds directly at any of the Th17 lineage genes because a Casz1 antibody that can be employed for CHIP purification is currently not available. Taken together, we have shown that while Casz1 can modulate Th1 and T_reg_ cell differentiation, only in certain cytokine contexts *in vivo*, it plays a central role in integrating cytokine cues and promoting Th17 differentiation program *in vitro* and *in vivo*. Control of Th17 program by Casz1, partially requires its ability to induce epigenetic changes in iTh17 genes. Thus, Casz1 signaling pathway represents an additional pharmacologically tractable signaling axis for modulating Th17 development and targeting a variety of immunodeficiency and inflammatory disorders.

## Ethics Statement

Experiments were performed at Case Western Reserve University under approved protocols in compliance with the Institutional Animal Care and Use Committee’s guidelines. Some replicate experiments, including EAE studies were done at NIAID, NIH under an approved protocol, and in compliance with the NIAID Institutional Animal Care and Use Committee’s guidelines.

## Author Contributions

PP designed the study, performed experiments, analyzed the data, and supervised the project. PP wrote, and AW and CT edited the manuscript. NB performed *in vitro* experiments, infections, and analyzed ELISA and qPCR data. LZ scored the EAE mice in a blinded fashion, isolated tissues, and contributed to discussions. PZ, LD, and SS generated Casz1 floxed mice and bred them in NEI mouse facility before transferring to PP. CT, ZL, CY, and YH performed RNA-seq experiments, analyzed the data, and contributed to discussions. RH, VB, and BH performed some of the replicate qPCR, ELISA, and microscopy experiments. FY and NB performed the ChIP experiments.

## Conflict of Interest Statement

The authors declare that the research was conducted in the absence of any commercial or financial relationships that could be construed as a potential conflict of interest.
