# Supplementary material for: Identification of Casz1 as a Regulatory Protein Controlling T Helper Cell Differentiation, Inflammation, and Immunity
Source: Front Immunol. 2018 Feb 7;9:184. doi: 10.3389/fimmu.2018.00184 (PMC5808336; doi:10.3389/fimmu.2018.00184)
Supplement: Supplementary file 1 [file Presentation_1.PDF]

Supplementary figures

Fig. S1

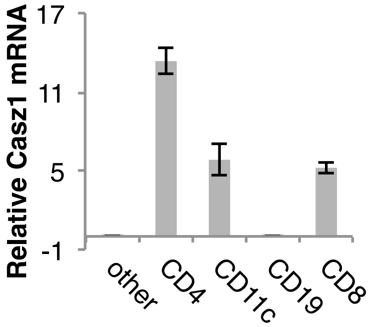

Fig. S1. Casz1 expression in immune cell subsets. Total $CD4^+$ T cells, $CD8^+$ T cells , $CD19^+$ B cells and $CD11c^+$ dendritic cells were positively sorted from mouse spleen and lymph nodes using PE-antibody conjugates and Mouse PE isolation kit from Stem Cell technologies. The negative fractions from the sort were also pooled (other). RNA was isolated from fresh cells. Relative Casz1 mRNA expression normalized to naïve $CD4^+$ T cells is shown.

Fig. S2

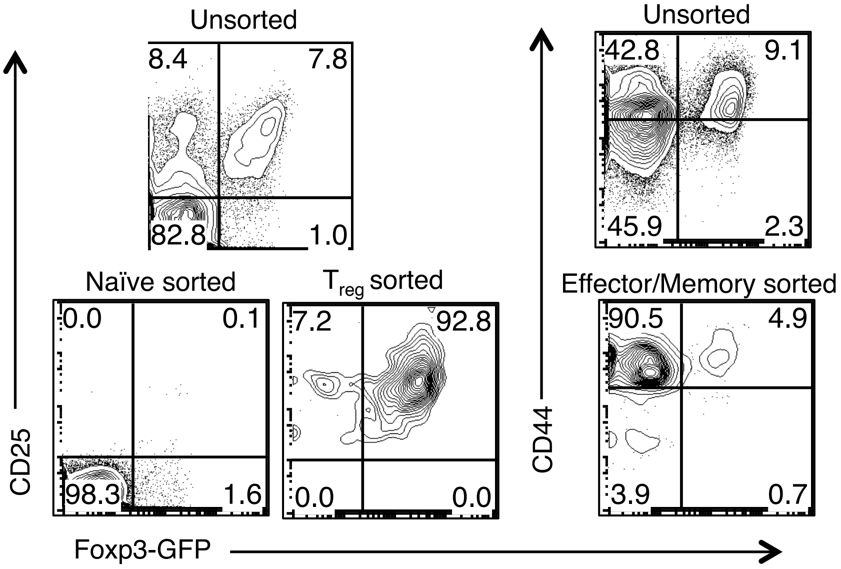

Fig. S2. Purity of various populations after sorting. $CD4^+$ T cells were FACS sorted from pooled lymph nodes and spleen, using CD4, CD25 and PE markers to obtain naïve and T_{reg} cells. Post-sort fractions were analyzed by flow cytometry. $CD44^+$ effector/memory cells were sorted from $CD4^+GFP^-$ fraction using CD44 biotin antibody and Anti-biotin purification kit from Stem Cell technologies. Sorted and unsorted fractions were stained with anti-CD44-percpCy5.5 antibody to assess the cell purity.

Fig. S3

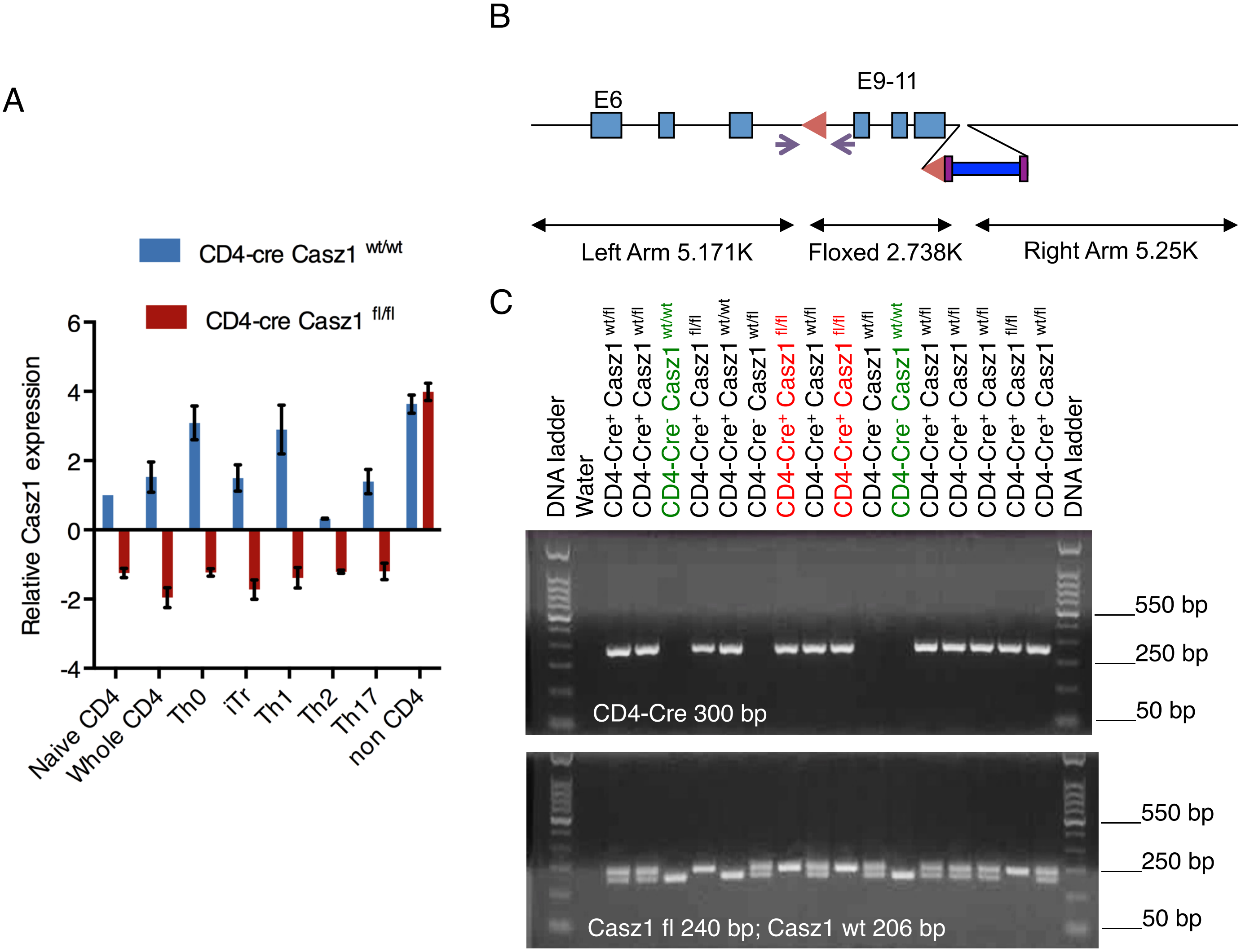

Fig. S3. A) Differential Casz1 mRNA expression in different CD4$^+$ cell subsets. CD4-cre Casz1$^{wt/wt}$ CD4-cre Casz1$^{fl/fl}$ naïve CD4$^+$ T cells were activated under indicated stimulation conditions (see Methods and Fig.S6). RNA was isolated on d3 after activation. Unstimulated whole CD4 T cells and CD4$^+$T cell depleted splenocytes (non CD4) were used as controls. Relative Casz1 mRNA expression normalized to naïve CD4+ T cells is shown. **B) Mouse *Casz1* (chr4:148,804,392-148,954,892) conditional targeting.** A BAC clone of SV129 origin spanning the entire locus was obtained from Source BioScience Life Sciences,UK (http://www.lifesciences.sourcebioscience.com). A LoxP site 159 bp upstream of exon (E)9 and another 135 bp downstream of exon 11 respectively were inserted so that a genomic region of 2.738 kb containing exons 9 through 11 are flanked by the two engineered LoxP sites. Removal of this genomic region upon Cre excision will lead to the deletion of 395 amino acid residues from the total of 1762 amino acids. Purple arrows indicate the positions of the genotyping primers **C) Genotyping gel showing CD4-cre and Casz1 floxed (fl) and wild-type (wt) alleles.** Genotyping PCR was performed with CD4-cre primers (Love Cre-fwd-5- CCT GGA AAA TGC TTC TGT CCG TTT G-3; Love Cre-rev-5- ACG AAC CTG GTC GAA ATC AGT GCG-3 (above),and CasZ1loxp5-fwd-5-GAA CTG TGG TTT CTG AGT CTT TGG AA-3 and CasZ1-loxp3-rev-5-CCA GAA AAG AAC ACC AAA CCA AGT TG-3 (below).

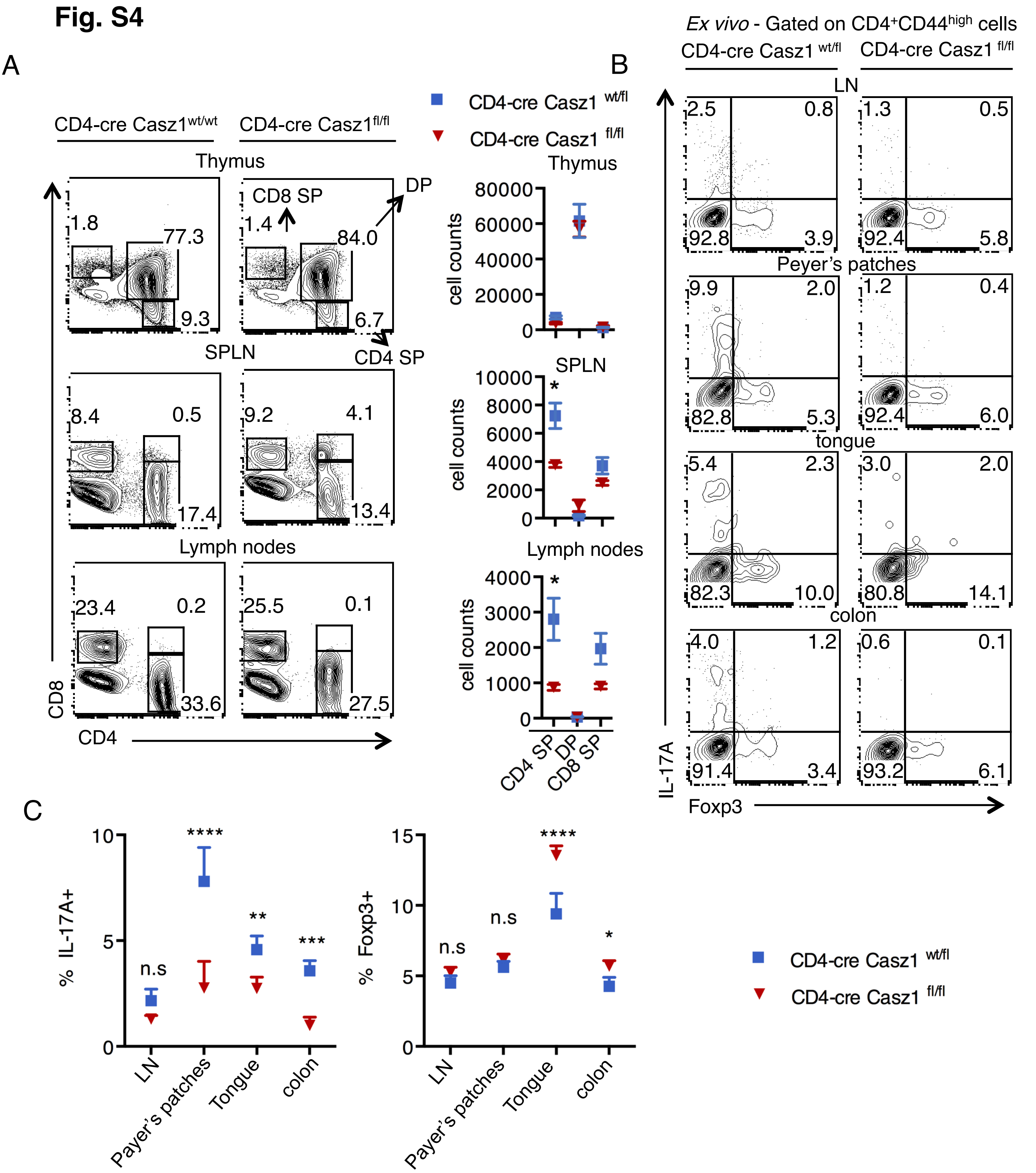

Fig. S4. T cell subsets in CD4-cre Casz1$^{wt/wt}$ and CD4-cre Casz1$^{fl/fl}$ mice. **A)** Frequency (left) and cell counts (right) of CD4 single positive (CD4 SP), CD4^{+}CD8^{+}double positive (DP) and CD8 SP cell populations in thymus, spleen and peripheral lymph nodes assessed *ex vivo*. **B) Memory Th17 cell frequencies are lower but memory T$_{reg}$ frequencies are higher in mucosal tissues of Casz1 deficient mice *ex vivo*. A)** Cells from pooled axillary, inguinal and cervical draining lymph nodes (LN), payers patches, tongues and the colon/small intestine (gut) were harvested from 6-8 week old mice and re-stimulated with PMA/ Ionomycin *ex vivo*. Flow cytometric dot plots of Foxp3 and IL-17A expression of CD4-cre Casz1 $^{wt/fl}$ mice (left), and CD4-cre Casz1 $^{fl/fl}$ mice (right panel). Plots show CD4^{+}CD44^{high} gated cells. **C)** Cells were stained as in (**B**). Data represent 3 mice/group and two independent experiments. (Statistical significance was determined by 2-way ANOVA test with 95% CI, alpha 0.05).

Fig. S5

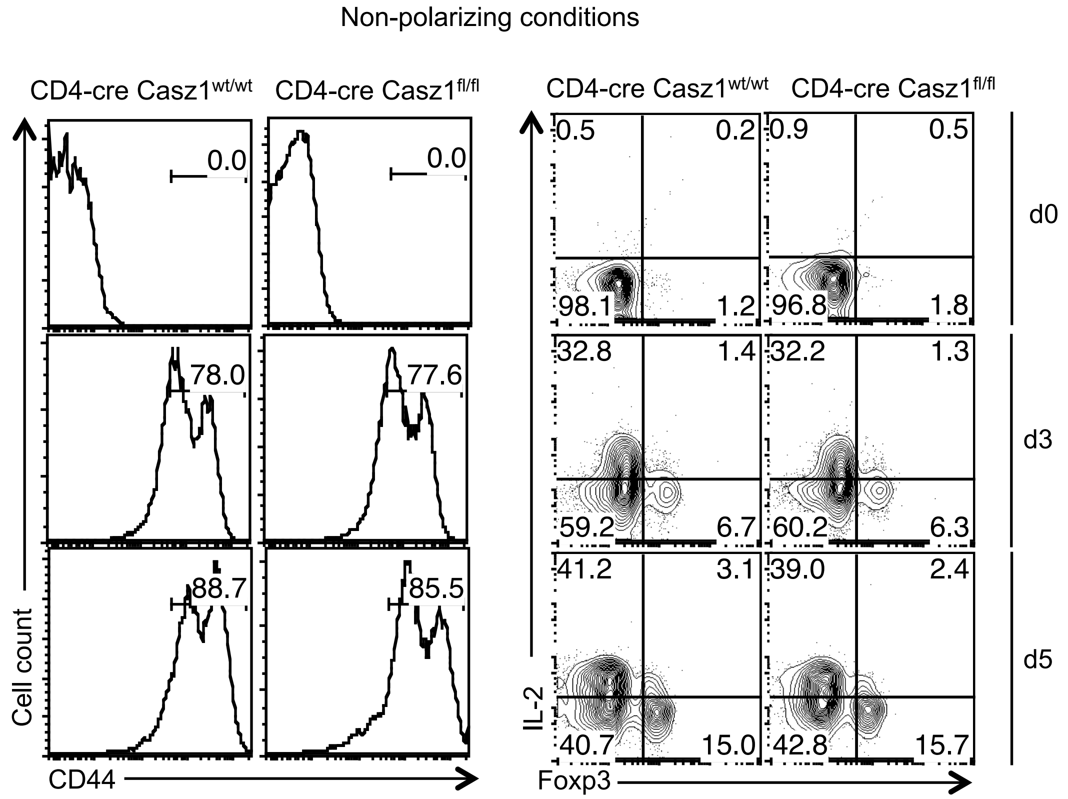

Fig.S5. Loss of Casz1 does not affect activation of CD4⁺ T cells under non-polarizing conditions *in vitro*. Flow cytometric contour plots showing CD44 (left), Foxp3 and IL-2 (right) expression at various time-points after activation (gated on CD4⁺ T cells). Cells were re-stimulated with PMA/Ionomycin for 4 hours to detect IL-2 expression.

Fig. S6

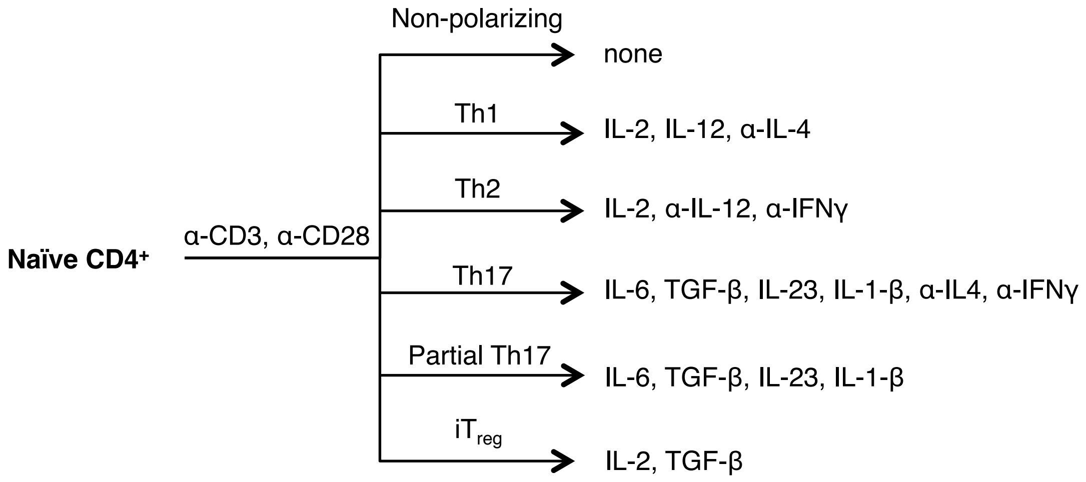

Fig.S6. Various polarization conditions used in the study. Naïve cells were stimulated with indicated cytokines and antibodies to achieve various skewing conditions *in vitro*.

Fig. S7

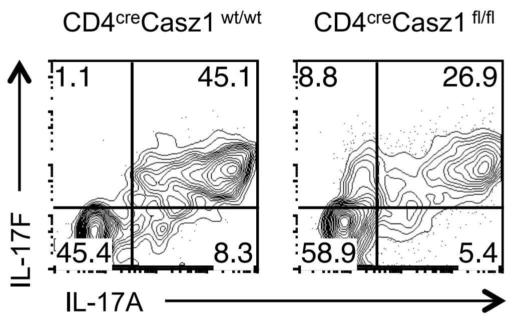

Fig.S7. Loss of Casz1 causes reduces the frequency of IL-17A and IL-17F co-expressing cells. Casz1$^{+/+}$WT (left) or Casz1$^{-/-}$ (right) naïve cells were stimulated under Th17 skewing conditions as in **Fig.3B** for 4 days, and were stained for IL-17A and IL-17F (gated on CD4 cells).

Fig. S8

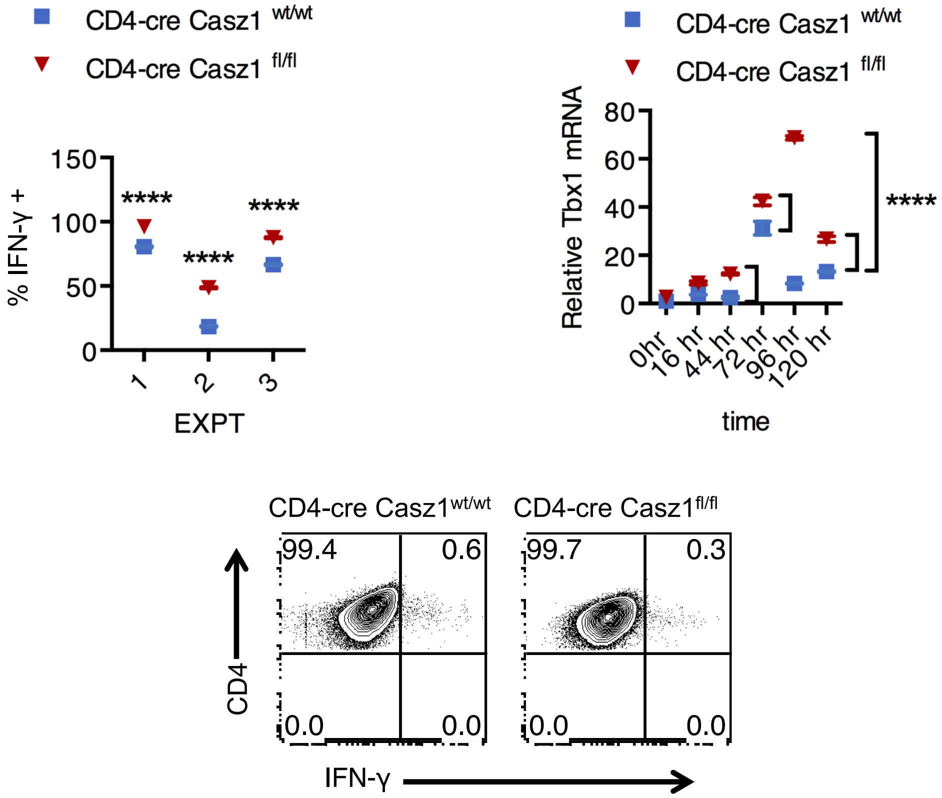

Fig.S8. Casz1 deficient cells show an enhanced Th1 phenotype in Th1 polarization conditions, but do not default to Th1 phenotype under Th17 polarization conditions. Casz1$^{+/+}$WT (left) or Casz1$^{-/-}$ (right) naïve cells were stimulated under Th1skewing conditions**(top panel)** as in **Fig.3A**, or Th17 skewing conditions **(bottom panel)** as in **Fig.3B** for 4 days , and were used for qPCR or stained for CD4 and IFN-γ (gated on CD4 cells). The frequency of IFN-γ+ cells from three independent experiments are shown (**top panel left**). qPCR was performed using Tbx1 primers (T-bet) in Th1 cells (**top panel, right**). 2-way ANOVA post hoc analysis was performed to determine the significance.

Fig. S9

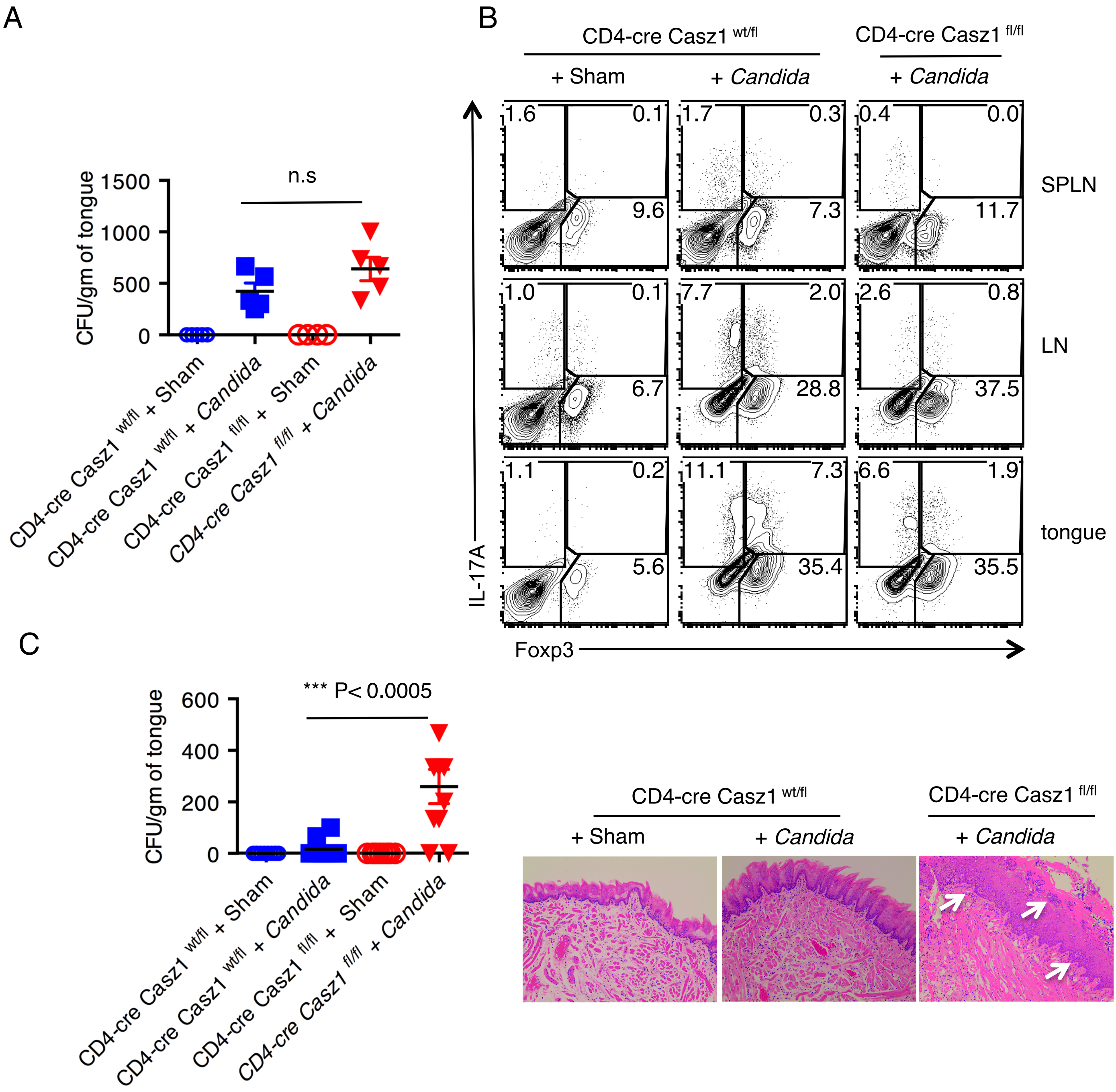

Fig. S9. Loss of Casz1 does not affect primary immune responses but reduce memory responses during OPC infection. **A)** 8-10 week old (n = 5/group) CD4-cre Casz1 $^{wt/fl}$ or Casz1$^{-/-}$CD4-cre Casz1 $^{fl/fl}$ were infected with sham control or *Candida* as described in Methods. On day 7 after infection, half of the tongues were lysed and plated on Sabaroud dextrose agar for 24 hours. Colony forming units (CFU) were counted. **B)** Mice were infected as in (**A**). They were re-infected on day 21. Spleen (SPLN), pooled axillary and cervical draining lymph nodes (LN) and the tongues were harvested and restimulated with PMA/ Ionomycin on day 2 after re-infection for Foxp3(X-axis) and IL-17A (Y-axis) staining (gated on CD4 cells). **C)** Mice were infected as in (**B**). CFU was measured on day 7 after reinfection (left). Histology sections of the tongue were stained with H&E (right). Microscopic images of the slides that were viewed at 200X magnification (arrows show infiltrating immune cells and inflammation). At least 5 independent experiments, comparing heterozygous with knockout animals, as well as WT with knockout animals showed similar results (*P< 0.05, as determined by Mann-Whitney tests).

Fig. S10

A

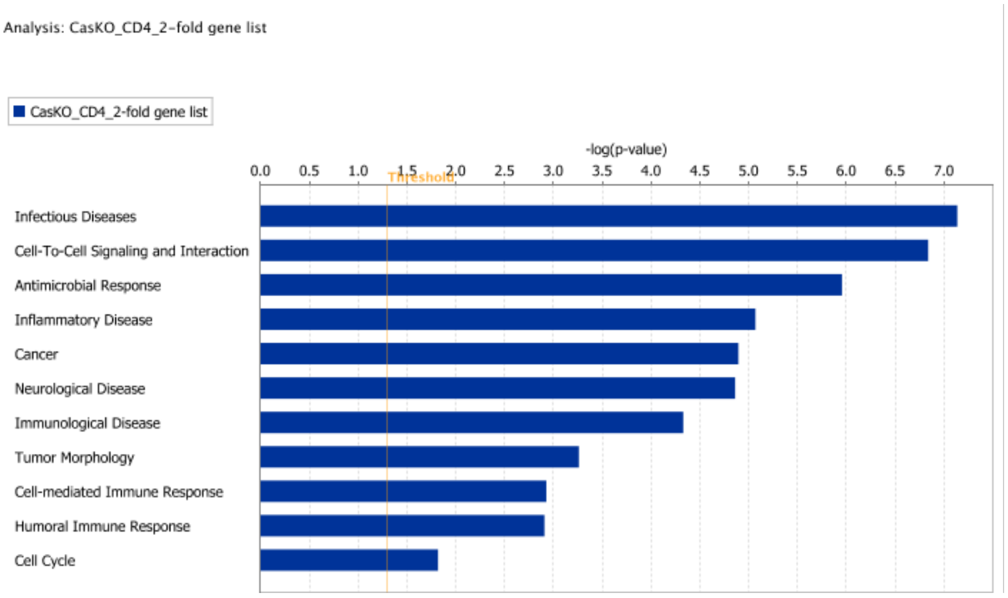

B

PROBE	RANK IN GENE LIST	RANK METRIC SCORE	RUNNING ES	CORE ENRICHMENT
NA.no	13	21.29730034	0.080021806	No
CASP1	52	13.55903435	0.12839484	No
KLF4	1130	2.978200674	0.046503745	No
IL6	1131	2.974989176	0.05783909	No
IL1B	1486	2.478215694	0.03663492	No
EZH2	2309	1.738303661	-0.027904442	No
HIF1A	3705	0.90070039	-0.14524135	No
SOX5	5537	0.017527346	-0.30368897	No
IL6R	6831	-0.482532293	-0.41378877	No
CMIP	8205	-1.11649251	-0.5283989	No
RUNX3	8531	-1.280381322	-0.5516564	No
PCGF2	10608	-3.116231203	-0.71950763	Yes
NOTCH1	10694	-3.294113874	-0.714315	Yes
STAT3	10756	-3.442255974	-0.7064802	Yes
IL23R	10869	-3.829170942	-0.70158637	Yes
FOSL2	11139	-5.135969162	-0.7053053	Yes
CCL20	11272	-5.993331432	-0.693897	Yes
IKZF3	11282	-6.07192421	-0.67154086	Yes
CCR6	11312	-6.423782349	-0.6495755	Yes
AHR	11313	-6.443375587	-0.62502486	Yes
BATF	11317	-6.482985497	-0.600583	Yes
IL1R1	11353	-7.029194832	-0.5768303	Yes
IRF4	11389	-7.699967861	-0.55052185	Yes
STAT5A	11429	-8.739269257	-0.52059966	Yes
IL21	11477	-10.41221714	-0.48499584	Yes
IL1RN	11533	-13.16948605	-0.4395788	Yes
RORA	11560	-18.23694611	-0.37234303	Yes
IL17F	11565	-20.6430912	-0.29403475	Yes
IL22	11568	-21.75301933	-0.21132427	Yes
RORC	11576	-26.49949265	-0.110961586	Yes
IL17A	11578	-29.21299553	2.60E-04	Yes

Fig. S10. A) Pathway analysis from RNA-seq data. Pathway analysis of the 11 molecular and immune functions most significantly dysregulated in CD4-cre Casz1$^{fl/fl}$ Th17 cells relative to their regulation in CD4-cre Casz1$^{wt/wt}$ Th17 cells , ranked by *P* value from most significant (top) to least significant (bottom); Results are presented as raw *P* values. Data are representative of one experiment with three independent biological replicates per group. **B) Literature curated Th17 signature genes used in GSEA (associated with Fig.6D).**

Supplementary Experimental Procedures

Mouse *Casz1* (chr4:148,804,392-148,954,892) conditional targeting

A bacterial artificial chromosome (BAC) clone of SV129 origin spanning the entire locus was obtained from Source BioScience Life Sciences,UK (http://www.lifesciences.sourcebioscience.com). A LoxP site 159 bp upstream of exon (E) 9 and another 135 bp downstream of exon 11 respectively were inserted so that a genomic region of 2.738 kb containing exons 9 through 11 are flanked by the two engineered LoxP sites. Removal of this genomic region upon Cre excision will lead to the deletion of 395 amino acid residues from the total of 1762 amino acids which accounts for the longest transcript of the gene (isoform 1; http://genome.ucsc.edu). This deletion will affect both isoforms 1 and 2 and is expected to leave downstream coding sequences out of frame. To place the two LoxP sites precisely at the designated locations, the approach of ET-recombination (or Recombineering) was used[1]. Briefly, the upstream LoxP site was introduced by insertion of a pre-constructed Neomycin (Neo)/Kanamycin (Kan) cassette flanked by LoxP sites (driven by the E. coli promoter gb2) through ET-mediated homologous recombination in the E. coli host of the BAC clone. Correctly recombined clones were enriched by Kan selection, identified by PCR reactions detecting recombination junctions, and subjected to next round of manipulation in which the inserted LoxP-Neo-Loxp cassette was removed by Cre excision leaving one LoxP site precisely the pre-designated location. This was achieved through transforming the E. coli host with a Cre- encoding plasmid in which the Cre expression is rendered under the control of the temperature sensitive E. coli promoter cI578 (λPR promoter)(GeneBridges, Heidelberg, Germany). Switching from 30°C to 37°C will activate the expression of Cre recombinase. After an overnight incubation at 37°C, clones were subjected to PCR screening identifying correctly recombined clones and subsequent verification by sequencing for the presence of the LoxP site at the desired location. In a similar fashion, the downstream LoxP site was inserted except that Neo)/Kan cassette used in this round of manipulation is also driven by PGK (Mouse phosphoglycerate kinase 1)[2] promoter and flanked with LoxP–Flippase recognition target (FRT) upstream and FRT only downstream[3]. The LoxP-FRT-Neo-FRT cassette is not removed as in the case of the first Neo cassette insertion so that the neo gene driven by the PGK promoter can be used as a positive selection marker in ES. Finally, the modified BAC clone containing appropriately positioned LoxP sites and the Neo selection marker flanked by FRT sites was truncated to a more manageable size so that both upstream and downstream targeting arms were reduced to about 5 kb of homologous sequences to the target locus. This last step of manipulation was achieved through another round of ET recombination between the modified BAC and a recipient plasmid vector as described [4,5]. W4 ES cell line derived from the 129S6/SvEvTac from Taconic was used.

Primers

The following primer sequences were used for qPCR: β-actin: 5'- TGACAGGATGCAGAAGGAGA-3', 5'-GTACTTGCGCTCAGGAGGAG-3', FoxP3: 5'- TTCATGCATCAGCTCTCCAC-3', 5'-TGATCATGGCTGGGTTGTC-3', ROR-γt: 5'-AACAGGAACAAGTGGCCAAG-3', 5'-GGTAGCTGCCCATCTGAGAG-3', IL-17A: 5'-AAAGCTCAGCGTGTCCAAA-3', 5'-GCGCCAAGGGAGTTAAAGAC-3', IL-2: 5'-GCAGGATGGAGAATTACAGGA-3', 5'-TTCAATTCTGTGGCCTGCTT-3', mouse Casz1: 5'-AGCTCATCAACGATGGCTTC-3', 5'TGCAGTGGAAGTGTGTGGTC-3', human Casz1: 5'-GCCTCCAAGTACGACTTCTTCA-3', 5'-GGATGGCTTCTTGTAGGTGCT-3'. The following primer sequences were used for CHIP PCR: *Il17a* promoter: 5'-AACAGTTGCGGTACTCAG-3', 5'-GCCCAAAGAAACCCACTCAATG-3', *Rorc*+2.7: 5'-TCAGAACTCAGCAGCCACAATAG-3', 5'-TGGCTCTGCCACCACAAAG-3', *Ahr* promoter: 5'-AACAGGTACTGGGCGAACAC-3', 5'-CTCCGCAGCCGCGACCCCGC-3', *Runx1* promoter: 5'-GCATCCGGGCTCAGCAGCAAGT-3', 5'-CTGTCC TGTGCGGATTTGGTGGC-3' and *IL17a CNS2B*: 5'-CAGCCCTGGTCCTTAAACTG-3', 5'- TCACTTTCG TTGTGCCTTTG-3'.

EAE scores

EAE scores were given according to the following chart: Score 0, healthy; Score 1, flaccid tail; Score 1.5, flaccid tail and impaired righting reflex; Score 2, impaired righting reflex and hind limb weakness; Score 2.5, one hind leg paralyzed; Score 3, both hind legs paralyzed with residual mobility in both legs; Score 3.5, both hind legs completely paralyzed; Score 4, both hind legs completely paralyzed and beginning front limb paralysis; Score 5, moribund or death of the animal after preceding clinical disease. Most mice reached the endpoint of our study based on their weight loss, (> 20% weight loss) when they reached the score of 3 - 4, and were euthanized according to our institutional guidelines.

Supplementary References

1. Zhang XM, Ng AH, Tanner JA, Wu WT, Copeland NG, Jenkins NA*, et al.* Highly restricted expression of Cre recombinase in cerebellar Purkinje cells. *Genesis* 2004, **40**(1)**:** 45-51.
2. Thomas KR, Capecchi MR. Site-directed mutagenesis by gene targeting in mouse embryo-derived stem cells. *Cell* 1987, **51**(3)**:** 503-512.
3. Chaiyachati BH, Kaundal RK, Zhao J, Wu J, Flavell R, Chi T. LoxP-FRT Trap (LOFT): a simple and flexible system for conventional and reversible gene targeting. *BMC biology* 2013, **10:** 96.
4. Muyrers JP, Zhang Y, Benes V, Testa G, Rientjes JM, Stewart AF. ET recombination: DNA engineering using homologous recombination in E. coli. *Methods in molecular biology* 2004, **256:** 107-121.
5. Testa G, Vintersten K, Zhang Y, Benes V, Muyrers JP, Stewart AF. BAC engineering for the generation of ES cell-targeting constructs and mouse transgenes. *Methods in molecular biology* 2004, **256:** 123-139.
